# Factors affecting medication adherence among older adults using tele-pharmacy services: a scoping review

**DOI:** 10.1186/s13690-022-00960-w

**Published:** 2022-08-31

**Authors:** Fatemeh Emadi, Arash Ghanbarzadegan, Sulmaz Ghahramani, Peivand Bastani, Melissa T Baysari

**Affiliations:** 1 Health and Clinical Sciences Unit, The University of South Australia, Adelaide, Australia; 2 Faculty of Health and Medical Sciences, The University of Adelaide, Adelaide, Australia; 3 Australian Research Centre for Population Oral Health, Adelaide Dental School, The University of Adelaide, Adelaide, Australia; 4 Menzies Centre for Health Policy and Economics, School of Public Health, Faculty of Medicine and Health, The University of Sydney, Sydney, Australia; 5 Health Policy Research Centre, Institute of Health, Shiraz University of Medical Sciences, Shiraz, Iran; 6 Faculty of Health and Behavioral Sciences, School of Dentistry, University of Queensland, Brisbane, Australia; 7 Biomedical Informatics and Digital Health, School of Medical Sciences, Faculty of Medicine and Health, The University of Sydney, Sydney, Australia

**Keywords:** Aged, Treatment Adherence and Compliance, Telemedicine

## Abstract

**Background:**

Medication adherence among older adults (aged 60 and above), particularly those with chronic conditions who take several medications, is critical, and tele-pharmacy services are a way to improve medication adherence. This study sought to determine the factors influencing medication adherence (MA) in older adults using tele-pharmacy services.

**Method:**

The Joana Briggs Institute scoping review methodology was implemented. Searches were conducted in databases PubMed, Scopus, ProQuest, Web of Science, and Embase from 2000 to the present day, to identify both qualitative and quantitative studies focusing on the use of tele-pharmacy by older people. Factors impacting MA were extracted and analyzed into themes using a qualitative approach. A concept map was also designed summarising these factors.

**Results:**

Of 7495 articles obtained in the initial search, 52 articles met the inclusion criteria. The analysis resulted in 5 themes and 21 sub-themes representing factors that impacted MA with tele-pharmacy. These themes are divided broadly into technology and user related factors. Technology factors included design of the tele-pharmacy intervention, commercial aspects, and adherence measurement method. User factors included user-health constraints, behaviors and perceptions.

**Conclusion:**

Industry, policymakers, and stakeholders should consider using tele-pharmacy services for improving medication adherence among older adults; however, ensuring interventions facilitate communication between patients and health care teams, and are accompanied by user training and support, is essential for technology uptake and effectiveness.

**Supplementary Information:**

The online version contains supplementary material available at 10.1186/s13690-022-00960-w.

## Background

Nonadherence to medications, particularly in older people, is associated with an increased risk of hospitalization and death [1] and a significant financial burden on the health care system [2]. Moreover, poor medication adherence (MA) is one of the strongest predictors of all-cause mortality in patients with chronic diseases such as heart failure and diabetes [3, 4]. Nonadherence to medications among older people is not uncommon, with a study of medication adherence among patients aged > 60 years revealing that less than 50% of people were adherent to medications [5]. The drivers for nonadherence are multi-factorial, with the World Health Organization (WHO) acknowledging that it is not only the patients themselves that contribute to poor adherence but that health service providers also play a role in improving MA [6].

The shortage of health professionals and the closure of pharmacies in remote areas is a growing problem worldwide that health systems must address to improve medication adherence among patients [7]. These issues are challenging in older patients (those over 60 years) due to their limited mobility and time and distance constraints, which may result in a reluctance to consume their medications at the appropriate time [8].Older adults are more likely to suffer from chronic diseases and take multiple medications daily (polypharmacy) than younger patients, necessitating regular and quick access to medications [9]. Also, the high prevalence of cognitive impairments among older people makes MA more challenging for this population [10]. The use of new technology, such as tele-pharmacy, could be a way to address some of these access barriers and improve MA.

The term tele-pharmacy describes the use of information and communication technology to deliver the components of pharmacy practice, including support to clinical services, remote education, patient counselling and monitoring, dispensing prescriptions and reconciliation of drug therapies. Tele-pharmacy is not a new initiative. Following the closure of several rural pharmacies, the first practical implementation of technology facilities in pharmacy service began in the early 2000s in North Dakota (USA). In recent years, the uptake of tele-pharmacy has increased exponentially, with strong support from relevant associations like the American Association of Health-System Pharmacists (ASHP) [2]. Despite the growing interest and uptake in tele-pharmacy research in recent years, no studies have comprehensively studied the facilitators and barriers of using tele-pharmacy services in older adults and the technology's impact on MA. The current scoping review investigated the key factors that affect the MA in older adults using tele-pharmacy interventions by analyzing papers reporting tele-pharmacy experiences, particularly in older patients. In addition, the benefits and challenges of tele-pharmacy for older people were examined to provide policymakers with enough information to make critical implementation decisions regarding the use of tele-pharmacy in this population.

## Methods

The present scoping review was conducted based on the JBI manual of evidence synthesis in 9 steps, as shown in Table 1 [11].

**Table 1:** Scoping review methodological steps

**Step**	**Title**	**Description**
**1**	Determination of the study question and objective	What is the impact of telepharmacy on medication adherence in older people?
**2**	Determination of the inclusion criteria	Defining the participants (older adults aged over 60 years), concept (medication adherence), and context (tele-pharmacy interventions)
**3**	Description of the search strategy	Defining the related keywords and their synonyms (Table 3, Additional file 1)
**4**	Evidence base searching	Searching the scientific databases including PubMed, Scopus, ProQuest, Web of Science, and Embase using related keywords
**5**	Evidence selection	Reviewing the articles and excluding papers, as shown in the PRISMA Fig. 1
**6**	Data extraction	Completing the extraction form including name, authors, publication year, study place, study design, key findings
**7**	Charting the data	Displaying the characteristics of the included studies
**8**	Summarizing data	Determining the main themes and sub-themes from the included studies
**9**	Consult with experts	Three experts in pharmacy and public health reviewing the findings
**10**	Trend Analysis	Illustration of the trend of tele-pharmacy interventions in different locations by the time

The Participants, Concept and Context (PCC) were defined as follows: the population was adults older than 60 years old. The concept included medication adherence in older adults, and the context included all tele-pharmacy services. All full-text articles and conference papers reporting a qualitative or quantitative study of tele-pharmacy services in older people were included. Review papers were excluded. The search strategy was designed around the three keywords: older people, medication adherence, and tele-pharmacy (Table 3, Additional file 1). Non-English papers were excluded on the title and abstract review. We included articles from 1^st^ January 2000 to 10^th^ August 2021 to capture any studies of the initial implementation of tele-pharmacy in the US.

**Fig. 1:**
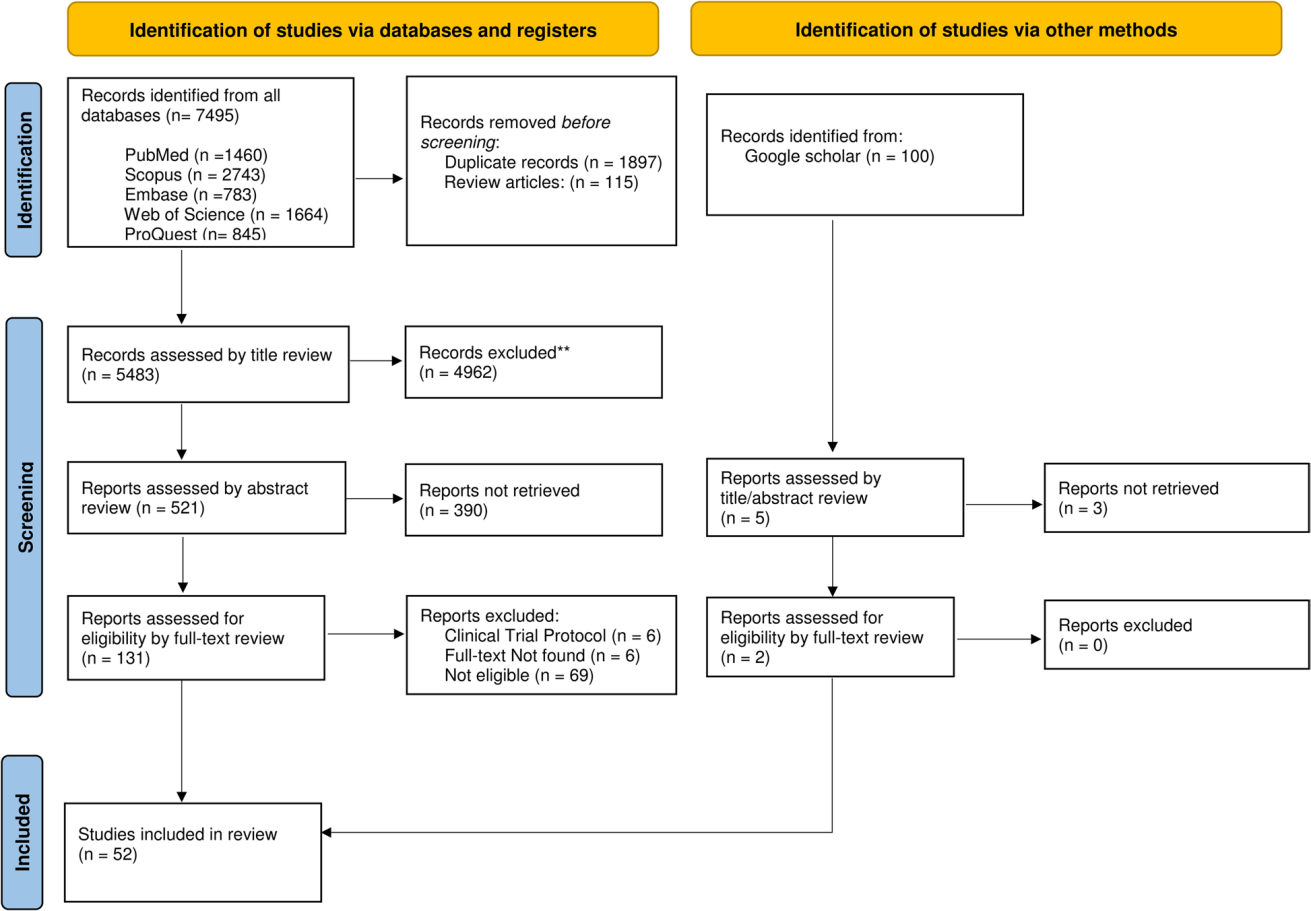
PRISMA flow chart of study selection

The systematic search and screening process results are shown step by step in Fig. 1 [12]. The search strategy was applied to five databases, including PUBMED, SCOPUS, ISI Web of Science, PROQUEST, and EMBASE. The first ten pages of Google Scholar were also hand searched for any grey literature. After removing duplicates, FE and AG independently reviewed all titles and abstracts, and the full-text of relevant articles was retrieved. Another researcher (PB) checked the articles based on the inclusion criteria and the study questions to ensure the included articles were eligible. The result of the systematic search has been shown step by step in Fig. 1. using PRISMA [12]. As described in step 6 of Table 1, the data were extracted and subsequently charted descriptively (Table 4, Additional file 2).

In several steps, extracted data were analyzed using a qualitative thematic analysis via the Thomas method. First, the extracted data were reviewed multiple times. Next, data units that best fit the study focus were highlighted and designated as initial codes that were evaluated and integrated to achieve the final codes. Subsequently, coding was done again to categorize the sub-themes per the study's purpose; this process helped identify key themes from sub-sub-themes, which were displayed in a table [13]. MAXQDA software (VERBI, MAXQDA 2020. Berlin) was used to analyze the data.

A thematic network was created applying VISIO plan software (Microsoft Corporation, 2018. Microsoft Visio), and the final map was reviewed by three experts in public health and pharmacy. The results were presented and discussed via the virtual panel to ensure the comprehensiveness and robustness of the results and accuracy of the final map. Furthermore, a 10^th^ step was added, as recommended by Ghanbarzadegan *et al.* [14], to illustrate the included publications over time. In this step, the location and intervention of focus for the publications was mapped over time to determine how the tele-pharmacy interventions in different countries changed over time.

## Results

Initially, searches returned a total of 7495 articles for the title and abstract screening (Table 3, Additional file 1). Then, 1897 duplicate articles and 115 review articles were excluded and 5484 studies remained for title screening. 521 studies were retrieved after removing the irrelevant titles and proceeded to the abstract screening phase. During abstract screening, 131 studies were selected as eligible studies for full text screening. 50 studies met the criteria of the scoping review and were included in our study, and two additional studies were identified through grey literature, resulting in a total of 52 studies for data extraction (Fig. 1). The extracted data are presented in Table 4, Additional file 2. Thematic analysis of the included studies led to the extraction of 5 main themes, 21 sub-themes and eighteen sub-sub-themes (Table 2). All references are presented in Table 5 in Additional file 3, and the main themes are described below.

**Table 2:** Factors impacting medication adherence in older adults using tele-pharmacy services

Main Theme	Sub-Theme	Sub-Sub Theme
Design of tele-pharmacy intervention	Patient -health care professional interaction	---
	Intervention method	Electronic medical device
		Message
		Mobile application
		Postal mail
		Web application
		Telephone Call
		e-prescription
		Remote dispensing by a pharmacist
	Available user manuals	---
	Usability	---
	Device size	---
	Medicine storage capacity	---
Commercial and Market Aspects	Affordability	---
	Government subsidies	---
	Security and privacy	---
Adherence Measurement	Measurement method	---
	Measurement scale	Medication delay
		Non-adherence level
		Non-compliance level
		Day-covered
		Forgot
		Refill delay
		Adherence level
		Timing adherence
User Health Constraints	Disease	---
	Polypharmacy	---
	Age	---
	Disability	---
	Family member/caregiver	---
	Remoteness	---
User behavior and perceptions	Baseline adherence	---
	Patient behavior	---
	Patient preference	---
	Long-term use	---

### Design of the tele-pharmacy intervention

One critical factor that impacted MA in tele-pharmacy was the interaction between patient and health care professionals. Both patients and health care professionals, such as physicians, pharmacists, and nurses, were actively involved in the tele-pharmacy intervention process in nearly half of the studies that resulted in improved MA, highlighting the importance of patient-health care professional interaction in a tele-pharmacy intervention’s success [15–38]. Patients' desire to participate in an adherence intervention was also important. Various methods of remote communication were identified, including electronic medical device [18, 25, 39], mobile application [40–42], web application [27, 43], text message [23, 44, 45], postal mail [46], phone call [26, 47, 48], electronic-prescription (e-prescription) [34, 49] and remote dispensing [50]. Electronic medical devices used in studies included interventions like in-home electronic medication dispensing systems and electronic dosage forms like blisters, eye drops and sprays to improve the MA among older adults. In addition to electronic devices, e-prescription or remote dispensing by the pharmacist were investigated in some trials, but only one study reported an improvement in MA [34, 49, 50]. Patients tended to use mobile applications rather than other tools like the message, postal mail, or telephone calls. Furthermore, access to user manuals to guide patients using the tele-pharmacy services improved MA [29, 30, 41, 51]. Other design factors that impacted MA included device size, usability, and medicine storage capacity, which improved the utilization of the tools and improved MA in older patients [29, 34, 37, 41, 48, 51].

### Commercial and market aspects

The commercial and economic aspects played essential roles when investigating the utilization of a tele-pharmacy intervention. Patients mentioned the affordability of the intervention as a factor that facilitated use [42, 48]. Moreover, government subsidies improved patients' willingness to use the interventions, leading to better medication adherence [37]. However, there were some concerns about patients' health data privacy and security [37, 48].

### Adherence measurement

A tele-pharmacy intervention's success was impacted by the MA measurement method. For instance, in a study investigating the MA of older people with heart failure using a telemonitoring medication system (MedSentry), the device data indicated high MA rates (95-99%) in the intervention group versus the usual care group. However, the self-report data showed no significant improvement in the telemonitoring group compared to the control group [21]. Similarly, another study showed considerable improvement in MA via Medication Event Monitoring System (MEMS) in older patients with coronary heart disease when the device data was used, but not self-reported data [52]. Although the Morisky scale and the proportion of days covered were the most common scales to measure the MA [28, 44, 50, 53], other scales were considered an indicator of the MA in several studies such as medication delay [45], refill delay [24], timing adherence [18], non-adherence [47] and non-compliance level [32].

### User health constraints

The use of tele-pharmacy to improve MA in older people was impacted by a wide range of individual health variables. One of the most important factor was found to be the disease itself. For example, older patients with chronic diseases such as diabetes, hypertension, hyperlipidemia, chronic kidney disease (CKD), chronic obstructive pulmonary disease (COPD) and heart diseases were more committed to taking their medications, as were those with cancer [27, 29, 42, 43, 52] . In contrast, no significant MA improvement was observed in patients with HIV and pain [54, 55]. As a large proportion of older adults experienced the aforementioned chronic diseases simultaneously, polypharmacy was found to be a general characteristic among older people, which led to increased utilization of the MA tools and, as a result, improved MA outcomes. Surprisingly, in two studies on patients with several chronic diseases, older adults showed a higher MA rate than younger adults [42, 45]. While in another study on patients with hypertension, no significant MA change was reported in the older age group [56].

Notably, disability was examined in a few studies, limiting the utilization of technology tools in geriatrics tracking adherence [37, 39]. A few studies also examined the role of family members or caregivers in interventions to assist older people in using technology. However, there was no substantial impact on MA; the patients' awareness and knowledge about their medications improved [57]. Furthermore, training sessions conducted by caregivers resulted in higher intention to use a mobile app reminder in older patients [30]. Finally, patient remoteness was also identified as a factor impacting MA. One study showed that providing health care via text, video, or phone call to older people in remote rural clinics can improve MA [48].

### User behavior and perceptions

The last main theme identified was user behavior and perceptions, which included four sub-themes: baseline MA, patient behavior, preference, and long-term use of interventions. Notably, participants who initially maintained a high level of adherence before the tele-pharmacy intervention reported no significant change in their adherence following the use of the tele-pharmacy services [51, 57, 58]. For example, in a study evaluating the effectiveness of mobile apps and electronic pillboxes on MA in older people, there was no significant change in MA, attributed to the very high levels of MA initially [59].

Several studies have demonstrated the importance of individual behavior in using the technology tools to improve MA [20, 23, 51]. For instance, Dugas et al. used the regulatory mode theory to test the role of locomotion and assessment to describe the effectiveness of mobile health (mHealth) tools among veterans with diabetes. They created DiaSocial, a mHealth tool to motivate veterans to take their medicine. In a 13-week pilot trial, low locomotion was linked to no change in MA, whereas high locomotion was linked to an improvement in MA rate [20]. A qualitative study identified four behavioral elements that enhance MA via tele-pharmacy service: commitment, awareness, feelings of involvement, and power to take medicine at the correct dose and time [23]. However, another study found no significant increase in MA despite patients' satisfaction with their increased awareness and commitment to taking medication [51].

In addition to individual behavior, patient preference was another sub-theme that emerged from included papers. In one study, patients were pleased with the smart pillbox bottle's usability. Still, they were dissatisfied with its size and functionality because they already used weekly pill organizers to manage their medications [34]. In another study, participants preferred mobile apps to text message reminders because of their greater interactivity [30], and in another study, patients preferred mobile apps over the electronic pill box [59].

The other important issue impacting MA was the long-term effect of the tele-pharmacy intervention. In several studies, MA improved in the short term; however, this improvement was not persistent over time, and people stopped using the tele-pharmacy intervention over time. Studies associated this decline with poor long-term commitment to the interventions [17, 31, 34]. For instance, following up on the older HIV patients using mobile app reminders for 48 weeks showed no change in MA [54], and in another study, cancer patients showed only short-term improvements in adherence to medications when using a mobile app [43]. In contrast, one study reported a higher MA rate with a six-month rather than the three-month intervention using a multimodal strategy, including an electronic pillbox and telephone counselling. Surprisingly, a study reported an adherence rate of 98.35% ± 2.15% over the 26 weeks follow-up [25].

#### Concept map of factors impacting medication adherence with tele-pharmacy

**Fig. 2:**
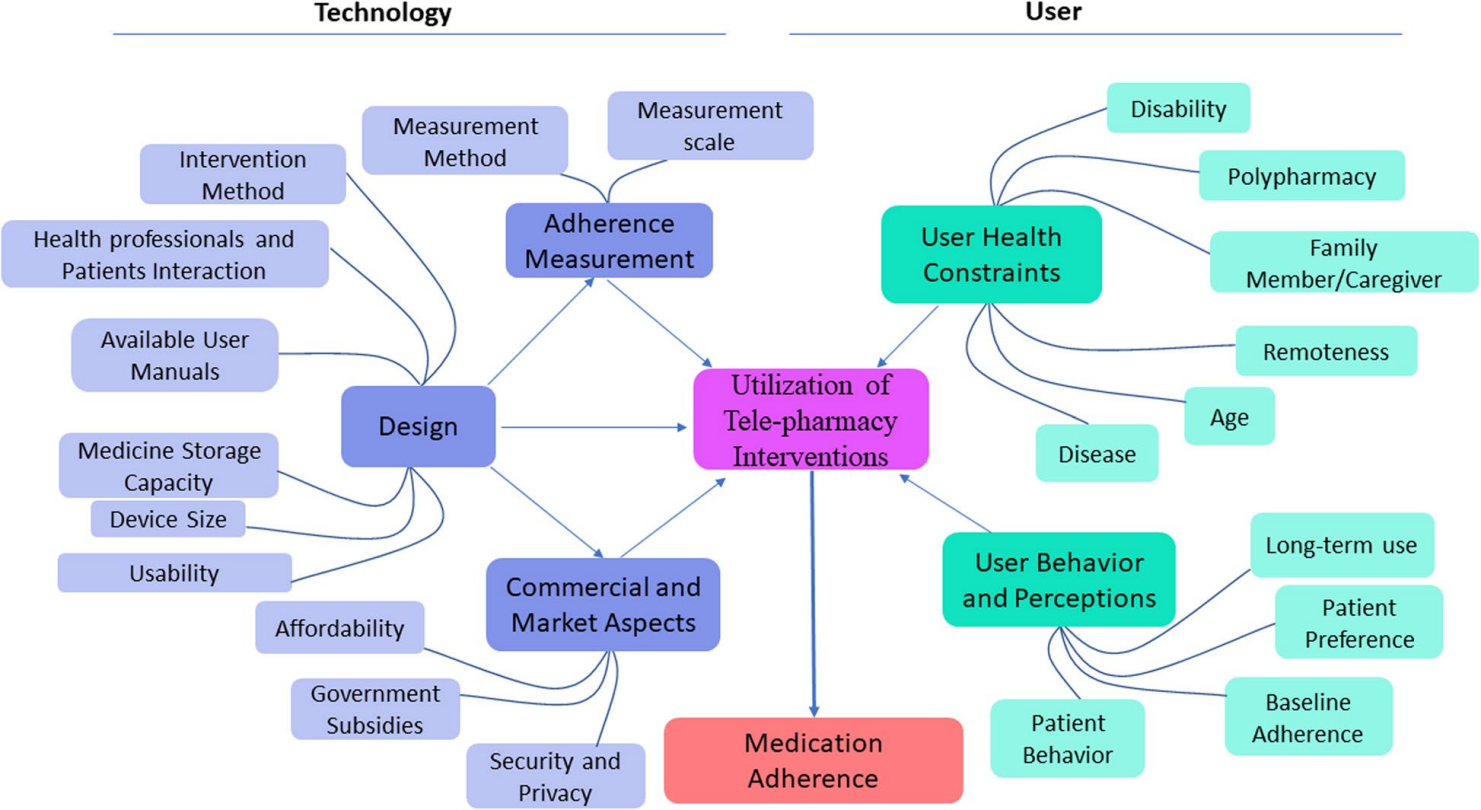
Concept map of factors affecting older adults’ medication adherence using telepharmacy interventions

The idea map depicted in Fig. 2 was built based on the major themes, the documented rational correlations in the included studies, and consensus among the study team. It conceptualizes the factors influencing MA in older adults using tele-pharmacy interventions. . The concept map is divided into two distinct sections: the technology and the user. The technology section consists of design, commercial and market considerations, and adherence assessment, whereas the user section consists of user-health restrictions and user behaviors and perceptions. The five major themes are connected with using tele-pharmacy tools to improve medication adherence in older people. Thus, the effect of tele-pharmacy services on older patients' medication adherence is influenced by their underlying sub-themes.

#### Trend analysis

**Fig. 3:**
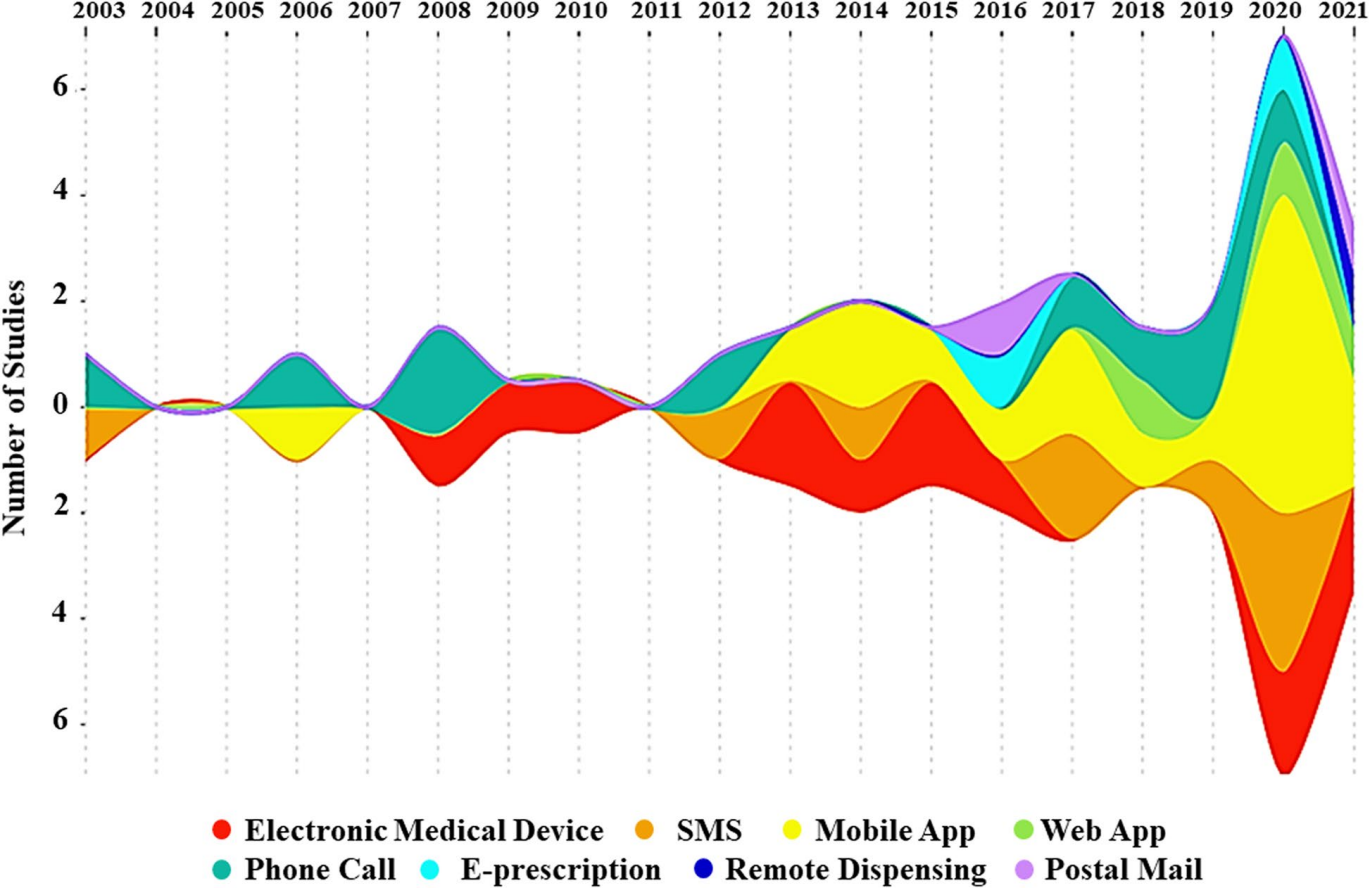
Stream graph of trend analysis (Intervention by publication year)

**Fig. 4:**
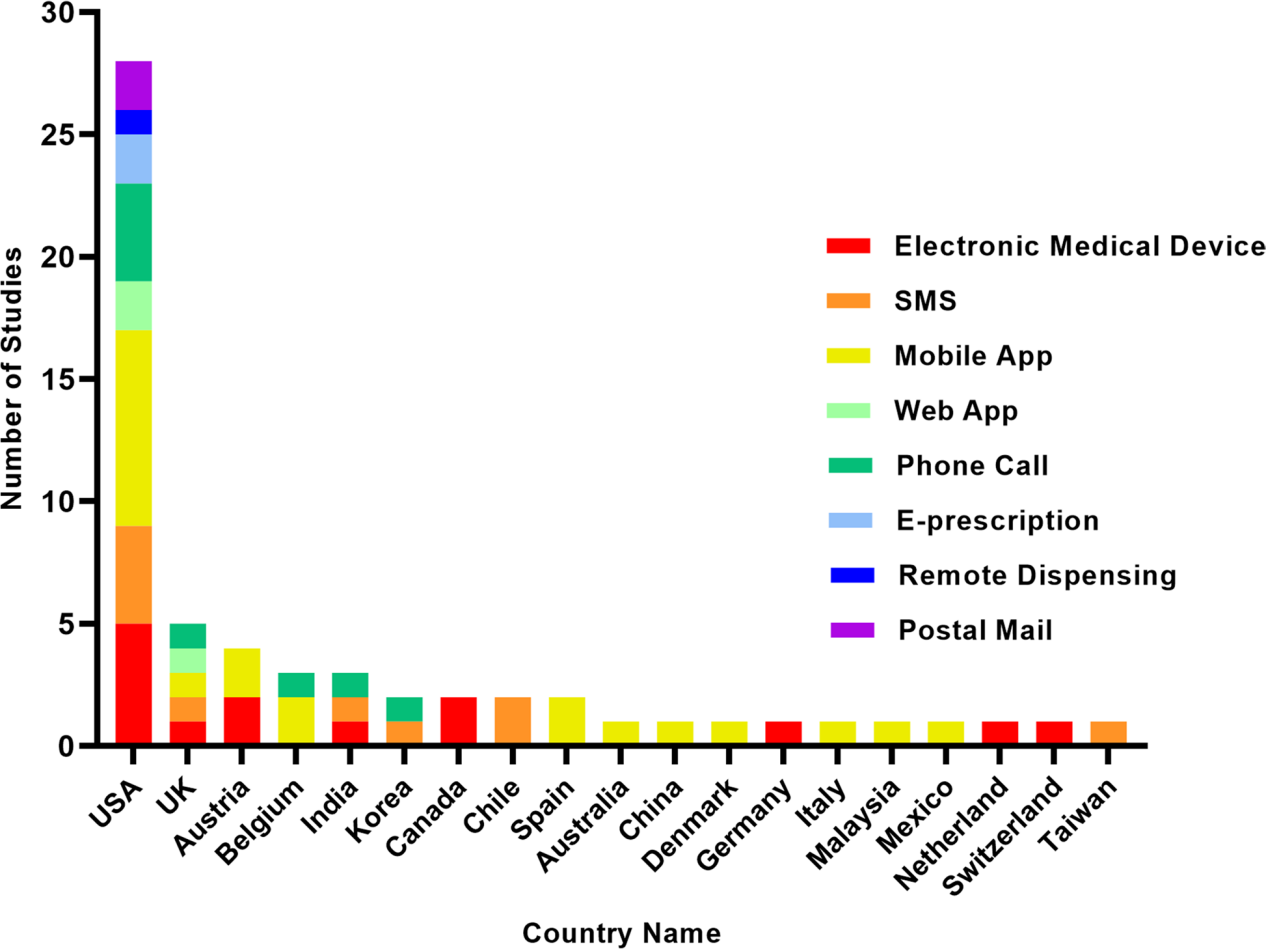
Bar chart of trend analysis (Publications count by location)

Figure 3 depicts the time trend of research on tele-pharmacy intervention approaches. Each color represents a distinct type of intervention in these streamgraphs, including in home electronic medical device, postal mail, mobile app, SMS, phone call, e-prescription, and remote dispensing by a pharmacist. As illustrated in Fig. 3, studies over time tended to focus on mobile application technology compared to other methods. Additionally, the majority of papers were published in 2019. As shown in Fig. 4, more than half of the studies included in our review were done in the United States. Additionally, American research has focused on various intervention strategies, whereas work in other countries has focused on a more limited number of intervention methods (Fig. 4).

## Discussion

This review identified several factors related to the technology and user that affects MA in older people using tele-pharmacy services. Overall, older patients, particularly those with chronic diseases, demonstrated good MA when using tele-pharmacy services. However, this effect might not be sustained. In several studies, the improvement in MA observed when using tele-pharmacy interventions was short-lived, and after a few months of using the service, MA declined. The one exception to this appeared to be medication dispensing systems (MDS), with a study showing that high MA was maintained for up to 26 weeks [25]. MDS include various features that may contribute to this sustained impact on MA, including reminders that alert users about medications and changes to symptoms and a feature that links patients with pharmacists and health care teams to help resolve any medication-related issues. However, studies also showed that the success of MDS was impacted by participants understanding the device functionalities, which highlights the importance of user support, training, and user manuals [29, 30, 41, 51].

An exciting result from this review was the key role that health professionals play in MA among older people, regardless of the tele-pharmacy technology used (text, phone call, app, device). This is likely because health professionals are a trusted source for patients and are often the first point of call for older people with a medication-related question or concern [60]. This suggests that tele-pharmacy interventions are likely more effective in supporting MA if they facilitate communication between patients and health professionals.

Our trend analysis showed that older patients are becoming more comfortable with using mobile applications over time. In addition, the majority of the included studies were completed between 2019 and 2021, indicating a recent surge in interest in this topic. Although there is a common perception that older people will not use technology for health, our review showed that this is rapidly changing, and in some cases, MA was higher in older groups (over 65 years) than in younger groups (less than 65 years) when using tele-pharmacy interventions [42, 45].

This review showed that many smartphone apps, particularly those that include interactive features, have been effective in improving MA for older patients. Although patients found text message reminders helpful in taking their medications, they preferred mobile applications because they were interactive and provided individualized health monitoring and personalized medication information [29]. Although mobile apps are gaining momentum, there remain issues that need to be adequately addressed to ensure apps are easy to use and useful for older people. For example, in one study, patients were unfamiliar with the generic name of medications in the application, and the design needed to be modified to auto-link generic and brand names to solve this problem [41]. Studies also showed that in the absence of strong support services and training sessions, older people could become confused when using apps [61].

In contrast to mobile apps, we found fewer papers focusing on other types of tele-pharmacy interventions. Although in recent years, e-prescriptions and remote dispensing by pharmacists are increasingly being used to improve medication adherence, only a small number of studies investigated the utilization of these tools in older people [34, 49, 50]. The bulk of studies in our review were also conducted in the USA, suggesting that other countries may be slower in adopting tele-pharmacy services. Our positive findings indicate high MA can be achieved with tele-pharmacy, and we recommend other nations, particularly those with a higher population of older people, consider these new technologies. Overall, tele-pharmacy services can significantly motivate patients to take their medications. Only a few trials reported poor MA after implementation of the intervention, and that is likely to be because of high baseline adherence prior to intervention [57–59], low numbers of participants [30, 58], and self-reported adherence measures, which are unreliable for determining the effectiveness of an intervention [21, 62].

There was no consistent approach used for measuring MA in studies, which made comparison difficult across the studies. Investigating medication adherence was not the primary purpose of some studies and MA was reported only as a secondary outcome. However, we included those studies as their outcomes aligned with the aim of the scoping review.

## Conclusion

In conclusion, tele-pharmacy can improve MA in older people, however, several factors impact the achievement of this goal. These factors relate to the intervention, including design, commercial aspects, and adherence measurement, and to the user, including health constraints behaviors, and perceptions. Communication with the health care team and sufficient user training appear necessary for tele-pharmacy interventions to enhance MA. In addition, intervention designers should focus on the design, size, storage capacity, and security of technologies. Government and policymakers should provide subsidies for such interventions to encourage uptake among older users. There is no one-size-fits-all strategy, and each patient's physical and mental characteristics should be considered when recommending the best treatment option, including tele-pharmacy interventions.

## Supplementary Information

**Additional file 1: Table 3.** Search Strategy.

**Additional file 2: Table 4.** Included articles extracted information.

**Additional file 3: Table 5. **Determinants of elders’ medication adherence in telepharmacy services with references.

## Data Availability

All data generated or analyzed during this study are included in this published article [and its supplementary information files]. Additional file 2 includes all included studies and extractions.

## Abbreviations

CKD: Chronic Kidney Disease
COPD: Chronic Obstructive Pulmonary Disease
E-prescription: Electronic Prescription
MA: Medication Adherence
MDS: Medication Dispensing System
MEMS: Medication Event Monitoring System
MHealth: Mobile Health
WHO: World Health Organisation
