# Supplementary material for: Factors affecting medication adherence among older adults using tele-pharmacy services: a scoping review
Source: Arch Public Health. 2022 Aug 31;80:199. doi: 10.1186/s13690-022-00960-w (PMC9429665; doi:10.1186/s13690-022-00960-w)
Supplement: Supplementary file 1 — Additional file 1: Table 3. Search Strategy. [file 13690_2022_960_MOESM1_ESM.docx]

**Table 3 - Search Strategy**

| Population | Concept | Context |  |
| --- | --- | --- | --- |
| Elders | Medication Adherence | Tele-Pharmacy |  |
| MeSH terms and Key Words | | | |
| Aged (MeSH) emtree | Treatment adherence and compliance (MeSH) | Electronic prescribing (MeSH) (emtree) |  |
| Older | Patient acceptance of healthcare (MeSH) | Telemedicine (MeSH) |  |
| Oldster | Patient compliance (MeSH)(emtree) | Tele-prescription |  |
| Elderly | Medication adherence (MeSH) | Digital prescription |  |
| Geriatrics (emtree) | Treatment adherence | electronic prescription(s) |  |
| Ageing population (AGING EMTREE) | Medication acceptability | Electronic pharmacy |  |
| Elder(s) | Medication persistence  Medication compliance(emtree) | Tele-pharmacy |  |
| Senior(s) | Drug compliance | E-prescription(s) |  |
| Frail elderly | Drug adherence | E-prescribing |  |
|  | Patient cooperation | Mobile health |  |
|  | Therapeutic adherence | Telehealth(emtree) |  |
|  |  | eHealth |  |
|  |  | mHealth |  |
|  |  | Electronic transmission of prescriptions |  |
|  |  | Digital pharmacy |  |
|  |  | Digital health |  |
| PubMed | | | |
| #1 AND #2 AND #3 | ("aged"[MeSH Terms] OR "older"[Title/Abstract] OR "oldster"[Title/Abstract] OR "geriatric"[Title/Abstract] OR "ageing population"[Title/Abstract] OR "elder*"[Title/Abstract] OR "senior*"[Title/Abstract]) AND "treatment adherence and compliance"[MeSH Terms] AND ("electronic prescribing"[MeSH Terms] OR "telemedicine"[MeSH Terms] OR "digital prescription"[Title/Abstract] OR "electronic pharmacy"[Title/Abstract] OR "telepharmacy"[Title/Abstract] OR "digital pharmacy"[Title/Abstract] OR "digital health"[Title/Abstract]) | |  |
| #1 | ((((((aged[MeSH Terms]) OR (older[Title/Abstract])) OR (oldster[Title/Abstract])) OR (geriatric[Title/Abstract])) OR (ageing population[Title/Abstract])) OR (elder*[Title/Abstract])) OR (senior*[Title/Abstract]) | |  |
| #2 | "treatment adherence and compliance"[MeSH Terms] | |  |
| #3 | ((((((((electronic prescribing[MeSH Terms]) OR (telemedicine[MeSH Terms])) OR (telepriscription[Title/Abstract])) OR (digital prescription[Title/Abstract])) OR (electronic pharmacy[Title/Abstract])) ) OR (telepharmacy[Title/Abstract])) OR (digital pharmacy[Title/Abstract])) OR (digital health[Title/Abstract]) | |  |
| Scopus | | | |
| #1 AND #2 AND #3 | ( ( TITLE-ABS-KEY ( aged ) OR TITLE-ABS-KEY ( older ) OR TITLE-ABS-KEY ( oldster ) OR TITLE-ABS-KEY ( elderly ) OR TITLE-ABS-KEY ( geriatric ) OR TITLE-ABS-KEY ( ageing AND population ) OR TITLE-ABS-KEY ( elder* ) ) ) AND ( ( TITLE-ABS-KEY ( adherence ) OR TITLE-ABS-KEY ( compliance ) OR TITLE-ABS-KEY ( cooperation ) ) ) AND ( ( TITLE-ABS-KEY ( electronic AND prescribing ) OR TITLE-ABS-KEY ( telemedicine ) OR TITLE-ABS-KEY ( teleprescription ) OR TITLE-ABS-KEY ( digital AND prescription ) OR TITLE-ABS-KEY ( electronic AND prescription ) OR TITLE-ABS-KEY ( electronic AND pharmacy ) OR TITLE-ABS-KEY ( telepharmacy ) OR TITLE-ABS-KEY ( eprescription ) OR TITLE-ABS-KEY ( eprescribing ) OR TITLE-ABS-KEY ( digital AND pharmacy ) OR TITLE-ABS-KEY ( digital AND health ) ) | |  |
| #1 | ( TITLE-ABS-KEY ( aged ) OR TITLE-ABS-KEY ( older ) OR TITLE-ABS-KEY ( oldster ) OR TITLE-ABS-KEY ( elderly ) OR TITLE-ABS-KEY ( geriatric ) OR TITLE-ABS-KEY ( ageing AND population ) OR TITLE-ABS-KEY ( elder* ) ) | |  |
| #2 | ( TITLE-ABS-KEY ( adherence ) OR TITLE-ABS-KEY ( compliance ) OR TITLE-ABS-KEY ( cooperation ) ) | |  |
| #3 | ( TITLE-ABS-KEY ( electronic AND prescribing ) OR TITLE-ABS-KEY ( telemedicine ) OR TITLE-ABS-KEY ( teleprescription ) OR TITLE-ABS-KEY ( digital AND prescription ) OR TITLE-ABS-KEY ( electronic AND prescription ) OR TITLE-ABS-KEY ( electronic AND pharmacy ) OR TITLE-ABS-KEY ( telepharmacy ) OR TITLE-ABS-KEY ( eprescription ) OR TITLE-ABS-KEY ( eprescribing ) OR TITLE-ABS-KEY ( digital AND pharmacy ) OR TITLE-ABS-KEY ( digital AND health ) ) | |  |
| Emabase | | |  |
| #1 AND #2 AND #3 | (('aged'/exp OR 'geriatrics'/exp OR 'aging'/exp) OR (older:ti,ab,kw OR oldster:ti,ab,kw OR elderly:ti,ab,kw)) AND (('patient compliance'/exp OR 'medication compliance'/exp) OR ('treatment adherence':ti,ab,kw OR 'treatment compliance':ti,ab,kw OR 'patient acceptance of healthcare':ti,ab,kw OR 'medication adherence':ti,ab,kw OR 'medication acceptability':ti,ab,kw OR 'medication persistence':ti,ab,kw OR 'drug compliance':ti,ab,kw OR 'drug adherence':ti,ab,kw OR 'therapeutic adherence':ti,ab,kw)) AND (('electronic prescribing'/exp OR 'telehealth'/exp) OR ('tele prescription':ti,ab,kw OR 'digital prescription':ti,ab,kw OR 'electronic prescription':ti,ab,kw OR 'electronic pharmacy':ti,ab,kw OR 'e prescription':ti,ab,kw OR 'e prescribing':ti,ab,kw OR 'mobile health':ti,ab,kw OR 'e health':ti,ab,kw OR mhealth:ti,ab,kw OR 'electronic transmission of prescription':ti,ab,kw OR 'digital pharmacy':ti,ab,kw OR 'digital health':ti,ab,kw)) | |  |
| #A | 'aged'/exp OR 'geriatrics'/exp OR 'aging'/exp | |  |
| #B | older:ti,ab,kw OR oldster:ti,ab,kw OR elderly:ti,ab,kw | |  |
| #1 | A OR B | |  |
| #C | 'patient compliance'/exp OR 'medication compliance'/exp | |  |
| #D | 'treatment adherence':ti,ab,kw OR 'treatment compliance':ti,ab,kw OR 'patient acceptance of healthcare':ti,ab,kw OR 'medication adherence':ti,ab,kw OR 'medication acceptability':ti,ab,kw OR 'medication persistence':ti,ab,kw OR 'drug compliance':ti,ab,kw OR 'drug adherence':ti,ab,kw OR 'therapeutic adherence':ti,ab,kw | |  |
| #2 | C OR D | |  |
| E | 'electronic prescribing'/exp OR 'telehealth'/exp | |  |
| F | 'tele prescription':ti,ab,kw OR 'digital prescription':ti,ab,kw OR 'electronic prescription':ti,ab,kw OR 'electronic pharmacy':ti,ab,kw OR 'e prescription':ti,ab,kw OR 'e prescribing':ti,ab,kw OR 'mobile health':ti,ab,kw OR 'e health':ti,ab,kw OR mhealth:ti,ab,kw OR 'electronic transmission of prescription':ti,ab,kw OR 'digital pharmacy':ti,ab,kw OR 'digital health':ti,ab,kw | |  |
| #3 | E OR F | |  |

**Web of science**

| #1 AND #2 AND #3 | (((((((((TS=(aged)) OR TS=(older)) OR TS=(oldster)) OR TS=(elderly)) OR TS=(geriatric)) OR TS=(aging population)) OR TS=(elder)) OR TS=(senior)) OR TS=(frail elderly)) AND ((((((((((((TS=(Treatment adherence and compliance )) OR TS=(Patient acceptance of healthcare )) OR TS=(Patient compliance )) OR TS=(medication adherence)) OR TS=(treatment adherence)) OR TS=(Medication acceptability)) OR TS=(Medication persistence)) OR TS=(Medication compliance)) OR TS=(Drug compliance)) OR TS=(Drug adherence)) OR TS=(Patient cooperation)) OR TS=(Therapeutic adherence)) AND ((((((((((((((((TS=(Electronic prescribing )) OR TS=(Telemedicine )) OR TS=(Tele-prescription)) OR TS=(Digital prescription)) OR TS=(electronic prescription)) OR TS=(Electronic pharmacy)) OR TS=(Tele-pharmacy)) OR TS=(E-prescription)) OR TS=(E-prescribing)) OR TS=(Mobile health)) OR TS=(Telehealth)) OR TS=(eHealth)) OR TS=(mHealth)) OR TS=(Electronic transmission of prescriptions)) OR TS=(Digital pharmacy)) OR TS=(Digital health)) |
| --- | --- |
| #1 | ((((((((TS=(aged)) OR TS=(older)) OR TS=(oldster)) OR TS=(elderly)) OR TS=(geriatric)) OR TS=(aging population)) OR TS=(elder)) OR TS=(senior)) OR TS=(frail elderly) |
| #2 | (((((((((((TS=(Treatment adherence and compliance )) OR TS=(Patient acceptance of healthcare )) OR TS=(Patient compliance )) OR TS=(medication adherence)) OR TS=(treatment adherence)) OR TS=(Medication acceptability)) OR TS=(Medication persistence)) OR TS=(Medication compliance)) OR TS=(Drug compliance)) OR TS=(Drug adherence)) OR TS=(Patient cooperation)) OR TS=(Therapeutic adherence) |
| #3 | (((((((((((((((TS=(Electronic prescribing )) OR TS=(Telemedicine )) OR TS=(Tele-prescription)) OR TS=(Digital prescription)) OR TS=(electronic prescription)) OR TS=(Electronic pharmacy)) OR TS=(Tele-pharmacy)) OR TS=(E-prescription)) OR TS=(E-prescribing)) OR TS=(Mobile health)) OR TS=(Telehealth)) OR TS=(eHealth)) OR TS=(mHealth)) OR TS=(Electronic transmission of prescriptions)) OR TS=(Digital pharmacy)) OR TS=(Digital health) |

**Proquest**

| #1 AND #2 AND #3 | (noft(aged) OR noft(older) OR noft(Oldster) OR noft(Elderly) OR noft(Geriatric) OR noft(Ageing population) OR noft(Elder) OR noft(Senior) OR noft(Frail elderly)) AND (noft(Treatment adherence and compliance ) OR noft(Patient acceptance of healthcare ) OR noft(Patient compliance ) OR noft(Medication adherence ) OR noft(Treatment adherence) OR noft(Medication acceptability) OR noft(Medication persistence) OR noft(Medication compliance) OR noft(Drug compliance) OR noft(Drug adherence) OR noft(Patient cooperation) OR noft(Therapeutic adherence)) AND (noft(Electronic prescribing ) OR noft(Telemedicine ) OR noft(Tele-prescription) OR noft(Digital prescription) OR noft(electronic prescription) OR noft(Electronic pharmacy) OR noft(Tele-pharmacy) OR noft(E-prescription) OR noft(E-prescribing) OR noft(Mobile health) OR noft(Telehealth) OR noft(eHealth) OR noft(mHealth) OR noft(Electronic transmission of prescriptions) OR noft(Digital pharmacy) OR noft(Digital health)) |
| --- | --- |
| #1 | noft(aged) OR noft(older) OR noft(Oldster) OR noft(Elderly) OR noft(Geriatric) OR noft(Ageing population) OR noft(Elder) OR noft(Senior) OR noft(Frail elderly) |
| #2 | noft(Treatment adherence and compliance ) OR noft(Patient acceptance of healthcare ) OR noft(Patient compliance ) OR noft(Medication adherence ) OR noft(Treatment adherence) OR noft(Medication acceptability) OR noft(Medication persistence) OR noft(Medication compliance) OR noft(Drug compliance) OR noft(Drug adherence) OR noft(Patient cooperation) OR noft(Therapeutic adherence) |
| #3 | noft(Electronic prescribing ) OR noft(Telemedicine ) OR noft(Tele-prescription) OR noft(Digital prescription) OR noft(electronic prescription) OR noft(Electronic pharmacy) OR noft(Tele-pharmacy) OR noft(E-prescription) OR noft(E-prescribing) OR noft(Mobile health) OR noft(Telehealth) OR noft(eHealth) OR noft(mHealth) OR noft(Electronic transmission of prescriptions) OR noft(Digital pharmacy) OR noft(Digital health) |
