# Supplementary material for: Factors affecting medication adherence among older adults using tele-pharmacy services: a scoping review
Source: Arch Public Health. 2022 Aug 31;80:199. doi: 10.1186/s13690-022-00960-w (PMC9429665; doi:10.1186/s13690-022-00960-w)
Supplement: Supplementary file 2 — Additional file 2: Table 4. Included articles extracted information. [file 13690_2022_960_MOESM2_ESM.docx]

**Table 4 Included articles extracted information**

| NO. | Authors | Year | Place | Population | Study Design | Intervention | Disease | Medication | Key Findings |
| --- | --- | --- | --- | --- | --- | --- | --- | --- | --- |
| 1 | Ajit R. R.; Fenerty, C. H.; Henson, D. B.(1) | 2010 | UK | 37 | observation cross sectional | eye drop dosing aid | Glaucoma | travoprost | eye drop electronic dosing aid can improve the MA |
| 2 | Ahmad A.; Chiu, V.; Arain, M. A.(2) | 2020 | Canada | 13 | qualitative | medication dispensing system | hypertension, diabetes, arthritis, anxiety, depression, asthma, sleep apnoea, chronic pain | polypharmacy | 1-MDS is useful for impaired patients2-MDS may not always be accurate in tracking adherence |
| 3 | Ammenwerth E.; Woess, S.; Baumgartner, C.; Fetz, B.; et al.(3) | 2015 | Austria | 25 | observation cross sectional | integrated telemonitoring surveillance system (device, app, NFC ID cards), Mycor (a multimodal intervention program) for education , self-monitoring with goal-setting and feedback, and regular clinical visit | coronary heart disease |  | Comparing telemonitoring phases 1 and 2 showed a decline in adherence rates regarding daily data transfers (drop from 86% to 77% ) which is comparable to other adherence studies |
| 4 | Antonicelli R.; Testarmata, P.; Spazzafumo, et al.(4) | 2008 | Italy | 57 | clinical trial | telephone weekly follow-up of taking medicines and health condition such as bp, hr, urine output and body weight | congestive heart failure | aldosterone receptor antagonists, beta- blocker, statins, and nitrate | Adherence to prescribed treatment was observed in 26/29 (89.7%) in the TM group versus 10/28 (35.7%) in the control group (p < 0.03 |
| 5 | Boeni F.; Hersberger, K. E.; Arnet, I.(5) | 2015 | switzerland | 1 | case report | pharmaceutical care service including counselling, electronic multidrug punch cards, feedback on electronic records | diabetes | Irbesartan, aspirin, atorvastatin, panto prazole, bisoporolol, clopidogrel, metformin, clindamycin, insulin lisprum and glargine | high adherence rate in both taking and timing adherence |
| 6 | Borah B. J.; Qiu, Y.; Shah, N. D.; Gleason, P. P.(6) | 2016 | USA | 201433 | observation cross sectional | postal mail for non-adherent patients | diabetes, hypertension, hyper-lipidemia |  | Physician mailing was associated with higher medication adherence in antidiabetic, statin, and RAS antagonist cohorts |
| 7 | Brath H.; Morak, J.; Kastenbauer, T.; Modre-Osprian, et al.(7) | 2013 | Austria | 53 | clinical trial | mobile heath app plus electronic blister | diabetes, hypertension, hyper-lipidemia | metformin, simvastatin, rosuvastatin, ramipril | improve MA in medication and positive attitude in participants |
| 8 | Chew S.; Lai, P. S. M.; Ng, C. J.(8) | 2020 | Malaysia | 5 | qualitative | Med assist app (medication reminder) |  |  | Cons: 1- Unfamiliar with generic names 2- Confusion with app terms 3- unaware about app features Pros: 1- Medication Summary page 2- Improve medication adherence/ Suggestion: 1- Auto link between drug name 2- Flexible alarm time 3- assistant in case of a problem |
| 9 | Criner G. J.; Cole, T.; Hahn, K. A.; et al. (9) | 2021 | USA | 138 | clinical trial | device with audio-visual reminder and smartphone app | COPD | Budesonide/Formoterol pMDI | 1- improve adherent day and dose. 2- more effective in aged>65 |
| 10 | Cynthia L. Russell, Sarah Owens, Karen Q. Hamburger, et al. (10) | 2010 | USA | 72 | qualitative | Medication Event Monitoring System (MEMS®) |  |  | older adult Renal Transplant recipients described mostly positive responses to using the MEMS and that perceptions of the device were not related to medication adherence. P |
| 11 | Desteghe L.; Kluts, K.; Vijgen, J.; Koopman, P. et al.(11) | 2017 | belgium | 15 | observation cross sectional | mobile app | heart failure | non-vitamin K antagonist anticoagulant | not effective as they already have a good adherence |
| 12 | Desteghe L.; Vijgen, J.; Koopman, P.; Dilling-Boer, D. et al. (12) | 2018 | Belgium | 48 | clinical trial | telemonitoring with telephone feedback | Heart failure | non-vitamin K antagonist anticoagulant | Although there was not much room for improvement, our study showed that regimen adherence increased with 4.1% during active telemonitoring and with 7.2% using additional direct feedback. |
| 13 | Dugas M.; Crowley, K. et al. (13) | 2018 | USA | 27 | clinical trial | mobile app clinician engagement | diabetes | not mentioned | no difference in MA between different study group |
| 14 | Elliott R. A.; Barber, N.; Clifford, S. et al. (14) | 2008 | UK | 205 | clinical trial | pharmacist telephone consulting | age >75 or have a chronic condition | not mentioned | Higher MA in elder adults/chronic condition with telephone consultation |
| 15 | Foreman K. F.; Stockl, K. M.; Le, L. B.; Fisk, E. et al. (15) | 2012 | USA | 290 | observation cross sectional | text message | chronic disease | anti-diabetic, beta-blocker, ... | 1-higher portion of days covered in intervention group versus cohort. 2- higher PDC in anti-diabetic and beta blockers |
| 16 | Forestal D. A.; Klaiman, T. A.; Peterson, A. M.; Heller, D. A. (16) | 2016 | USA | 148325 | observation cross sectional | e-prescription |  |  | e-prescription less claimed as a proxy of MA |
| 17 | Goldstein C. M.; Gathright, E. C.; Dolansky, M. A. et al. (17) | 2014 | USA | 58 | clinical trial | smartphone app and electronic pill box (both have reminder) | HF |  | 1- there was no significant difference in MA between the smartphone and pill box group. 2- active or passive reminder in both groups was not significantly associated with the MA |
| 18 | Graetz I.; McKillop, C. N.; Stepanski, E. et al. (18) | 2018 | USA | 44 | clinical trial | web-based app | breast cancer | aromatase inhibitor | Weekly reminders to use a web-based app to report AI adherence and treatment-related symptoms demonstrated feasibility and improved short-term AI adherence |
| 19 | Guadamuz J. S.; McCormick, C. D.; Choi, S.; et al. (19) | 2021 | USA | 10404 | observation cross sectional | tele pharmacy |  | statins, ACEs/ARBs, and NIDM (non-insulin diabetic medications) | medication adherence was lower among users of telepharmacy than users of a traditional pharmacy in some but not all drug classes examined |
| 20 | Hale T. M.; Jethwani, K.; Kandola, M. S.; et al. (20) | 2016 | USA | 25 | clinical trial | MedSentry remote medication monitoring system (tele-monitoring device and monitoring centre with advisors) | HF |  | Telemonitoring in HF patients leads to higher MA. |
| 21 | Holender A.; Sutton, S.; De Simoni, A. (21) | 2018 | UK | 12 | qualitative | Patients perception of using technology to improve MA |  | cardiovascular | cons: 1-unfamiliar with technology. 2- lack of accessibility to tech.3- expensive cost.4- tech reduces the communication. 5- concern about data security and privacy. 6- concern about dependence on other or tech itself. pros: 1-tech aids the memory through reminder.2- tech reduces the confusion with polypharmacy/ suggestions: wearable or ingestible sensors can be used for patients with impairment |
| 22 | Huang C. Y.; Nguyen, P. A. A.; Clinciu, D. L. et al. (22) | 2017 | Taiwan | 203 | clinical trial | SMS medication reminder |  |  | unless there was no significant difference in control and intervention group of the 65>, there was more improvement in both medication delay and forgot to take medication in >65 group comparing to other age groups |
| 23 | Kassavou A.; A'Court, C. E.; Chauhan, J.; Brimocombe, J. D.; et al. (23) | 2020 | UK | 18 | clinical trial | text messaging service and smartphone app | hypertension |  | increased MA through 4 ways: (a) by increasing participants’ commitment to reply to the query message, (b) by raising awareness of tablet-taking routine, (c) by increasing feelings of involvement with their own medication-taking routine, and (d) by empowering them to take their medications as prescribed |
| 24 | Kim M. (24) | 2019 | Korea | 124 | clinical trial | Long-Message Service and Phone-Based Health-Coaching | hypertension |  | using long text message and health coaching improves the MA in people over 65 years |
| 25 | Kobb R.; Hoffman, N.; Lodge, R.; Kline, S. (25) | 2003 | USA | 1401 | clinical trial | rural home care service (telephone, text, video message) | hypertension, diabetes, cardiac arrythmia, COPD, CHF |  | rural home care service via tele-health improve the medication compliance in older patients |
| 26 | Kooy M. J.; van Wijk, B. L. G.; Heerdink, E. R. et al. (26) | 2013 | Netherland | 399 | clinical trial | electronic reminder device with or without counselling |  | statins | no statistically significant improvement of refill adherence when an ERD was used with or without counselling. However, in a subgroup of women using statins for secondary prevention the ERD-improved adherence was statistically significant. |
| 27 | Lee Jung-Ah; Evangelista, Lorraine S.; Moore, Alison A. et al. (27) | 2016 | USA | 18 | clinical trial | mobile app |  | oral anti-coagulant | An important but frequently faced challenge in a study of this kind is the reliance on self-report outcome measures. More objective measures of medication adherence (e.g., medical record review and pharmacy records) to supplement self-reported measure can be used |
| 28 | Lien Desteghe; Kluts, Kiki; Vijgen, Johan; et al. (28) | 2017 | Belgium | 15 | clinical trial | health buddie’s app relationship with grandchildren | AF | non-vitamin K antagonist oral anticoagulants (NOACs) | Only one patient indicated that the app improved his adherence, although 40% of the patients became more conscious about strict medication adherence. Interestingly, the majority of the patients indicated that they already had very good adherence to their NOAC therapy, also reflected in the self-reported Morisky scale with a mean patient score of 7.7 (out of 8) at the start of the study. |
| 29 | Mira José Joaquín; Susana, Lorenzo; Nuria, Toro. (29) | 2014 | Spain | 99 | clinical trial | medication self-management app |  | multiple medications | app improves adherence |
| 30 | Mubashir Aslam Arain; Armghan, Ahmad; Chiu, Venus; et al. (30) | 2021 | Canada | 48 | clinical trial | In-home electronic medication dispensing system | Arthritis, diabetes, COPD, hypertension, Anxiety |  | The MDS can be an effective, long-term solution to medication non-adherence in older adults experiencing chronic conditions and taking multiple medications. |
| 31 | Ownby R. L.; Hertzog, C.; Czaja, S. J. (31) | 2012 | USA | 23 | clinical trial | Automated reminding system via telephone call |  |  | individuals in both intervention groups(automated telephone reminder and tailored information) had higher levels of medication adherence than those in the control group |
| 32 | Panuccio, Vincenzo; Morante, Salvatore; Villa, Antonino; et al.(32) | 2020 | USA | 22 | observation cross sectional | mobile and web application | CKD | anti-hypertensive | Mobile APP may improve adherence to therapy in patients affected by CKD through making patients more involved in the management of their clinical condition |
| 33 | Park, L. G. Howie-Esquivel, J. Chung, M. L. Dracup, K. (33) | 2014 | USA | 90 | clinical trial | Text message (TM) | coronary heart disease | anti-platelet, Statin | 2 methods used to assess the MA, MEMS and self-report. MEMS shows that TM improves the MA to anti-platelet and statins, while self-reports shows no significant effect. |
| 34 | Park Linda G.; Ng, Fion; K. Shim Janet; et al. (34) | 2020 | USA | 28 | qualitative | mobile app text message reminder | coronary heart disease |  | 1-participants perception: text message reminders are convenient, easy, and flexible tool to establish a routine for taking medications. 2- participants were eager to use applications for their greater interactivity, individualized health monitoring, and personalized medication information. 3- participants shared preferred features (i.e. drug interactions, tracking symptoms) |
| 35 | Park, Daniel Youngjoon (35) | 2019 | USA | 29 | observation cross sectional | reminder app training session |  |  | trained group reported higher proportion of active app use |
| 36 | Park H.; Adeyemi, A.; Wang, W.; Roane, T. E. (36) | 2017 | USA | 563 | quasi-experimental design | The reminder call services were offered to eligible MAPD beneficiaries, and they included a live interactive conversation with patients to assess the use of their medications. | hypertension | anti-hypertensive | Antihypertensive medication adherence increased in both reminder call and control groups, but the increase was significantly higher in the intervention group. A telephonic outreach program was effective in improving antihypertensive medication adherence |
| 37 | Patton, D. E. Pearce, C. J. Cartwright, M. Smith, F. et al. (37) | 2021 | UK | 60 | clinical trial | Web-application, |  | poly-pharmacy | the community pharmacist intervention leads to better MA in pilot study, however the result of the final study still has not published. |
| 38 | Puig J.; Echeverria, P.; Lluch, T.; Herms, J.; Estany, C.; et al.(38) | 2021 | Spain | 100 | clinical trial | routine medical care application | HIV | anti-retroviral | There were no significant changes in adherence at week 48 (p = 0.493). |
| 39 | Qvist I.; Lindholt, J. S.; Søgaard, R.; et al. (39) | 2020 | Denmark | 1446 | clinical trial | telephone counselling | abdominal aortic aneurysm (AAA), peripheral arterial disease (PAD) or high blood pressure (HB) | statins, antithrombotic or antihypertensive | TC 3 months after screening improved adherence to statin at 6-month follow-up, but had no effect on the composite treatment, statins, antithrombotic or antihypertensive treatment over 60 months of follow-up |
| 40 | Ramachandran B.; Trinacty, C. M.; Wharam, J. F.; et al. (40) | 2021 | USA | 63012 | clinical trial | Mail order pharmacy (MOP) including a mailed letter, secure email message, and automated telephone call | diabetes | metformin | improved MA to metformin using mail order pharmacy |
| 41 | Redfern J.; Coorey, G.; Mulley, J.; Scaria, A.; et al.(41) | 2020 | Australia | 934 | clinical trial | interactive app | cardiovascular | anti-hypertensive and lipid lowering | there was no significant change in MA of anti-BP or anti-lipid medicine and overall MA as well. |
| 42 | Sadaf Faisal ; Jessica Ivo 1 ; Aidan McDougall, et al. (42) | 2020 | Canada | 37 | qualitative | 23 electronic MA products including pill dispenser, reminders, apps, ... |  |  | 1-preference for a particular electronic medication adherence product depends on multiple factors, including, but not limited to, the storage capacity, security, cost, and size of the device. 2- health care providers should consider patient-related factors such as cognitive and functional capability to operate a device, medication regimens, and product features to choose the right product for the right patient. 3-The manufacturers of these electronic medication adherence products should also consider the involvement of users in the beginning stages of product development for these technologies to ensure high acceptability, user friendliness, and affordability for end users. 4-Policy makers should consider subsidizing the cost of electronic medication adherence products to make them affordable |
| 43 | Schmidt S.; Sheikzadeh, S.; Beil, B.; Patten, M.; Stettin, J. (43) | 2008 | Germany | 62 | observation cross sectional | data send by pill box, reminded by health professionals |  |  | self-reported noncompliance was significantly lower in study patients |
| 44 | Sheilini M.; Hande, H. M.; Prabhu, M. M.; Pai, M. S.; George, A. (44) | 2019 | India | 160 | clinical trial | multimodal intervention including phone call follow up and weekly pill box reminder | hypertension | anti-hypertensive | the intervention improves the MA by the time in intervention and control group but not significant change between the control and intervention groups. |
| 45 | Shukla G.; Tejus, A.; Vishnuprasad, R.; Pradhan, S.; Prakash, M. S. (45) | 2020 | India | 111 | observation cross sectional | SMS and Whats App text reminder | hypertension | anti-hypertensive | after intervention, 95 out of 111 patients (85.6%) more than 60 years old, had MA to anti-hypertensive medications |
| 46 | Sterns A. A.; Sterns, H. L.. (46) | 2006 | USA | 49 | observation cross sectional | RX reminder application |  |  | patient satisfaction about being more conscious and committed to getting pills but not significant effect on MA was observed |
| 47 | Toscos T.; Drouin, M.; Pater, J. A.; Flanagan, M.; Wagner, S.; et al. (47) | 2020 | USA | 160 | observation cross sectional | e-prescribing software and smart pill bottle | AF |  | 1-Patients maintained an average adherence rate of 90.0% according to the smart pill bottle. 2-Patients found the bottle easy to operate but suggested that its size and functionality did not fit seamlessly into their existing routine, as many used weekly pill organizers to manage multiple medications |
| 48 | Varleta P.; Acevedo, M.; Akel, C.; Salinas, C.; et al. (48) | 2017 | chile | 314 | clinical trial | SMS | hypertension |  | not significant in older patients |
| 49 | Wilkie D. J.; Yao, Y.; Ezenwa, M. O.; Suarez, M. L.; et al. (49) | 2020 | USA | 234 | clinical trial | Application, (PAINRelieveIt) | Pain | analgesic | no significant effect on the analgesic adherence |
| 50 | Wu J. Y. F.; Leung, W. Y. S.; Chang, S.; Lee, B.; Zee, B.; Tong, P. C. Y.; Chan, J. C. N.(50) | 2006 | china | 442 | clinical trial | telephone counselling |  | polypharmacy | Periodic telephone counselling by a pharmacist improved compliance and reduced mortality |
| 51 | Zárate-Bravo Ernesto; García-Vázquez, Juan-Pablo; Torres-Cervantes, Engracia; et al. (51) | 2020 | Mexico | 16 | clinical trial | Medication Ambient Display (MAD) including: mobile application including 1-Auditory and visual reminders and 2- Events that may enhance older adults’ awareness about whether the medication was taken and 3-repesentation of their medical adherence |  |  | using ambient modalities for implementing external cues is useful for drawing the attention of older adults to remind them to take medications and to provide immediate awareness on adherence behaviour. |
| 52 | Zhai P. P.; Hayat, K.; Ji, W. J.; Li, Q.; Shi, L.; Atif, N.; Xu, D.; Li, P. C.; Du, Q. Q.; Fang, Y. (52) | 2020 | China | 384 | clinical trial | SMS | hypertension |  | significant improvements in medication adherence |

**References for Table 4**

1. Ajit RRF, C. H.; Henson, D. B. Patterns and rate of adherence to glaucoma therapy using an electronic dosing aid. Eye. 2010;24(8):1338-43.

2. Ahmad AC, V.; Arain, M. A. Users' Perceptions of an in-Home Electronic Medication Dispensing System: A Qualitative Study. MEDICAL DEVICES-EVIDENCE AND RESEARCH. 2020;13:31-9.

3. Ammenwerth EW, S.; Baumgartner, C.; Fetz, B.; van der Heidt, A.; Kastner, P.; Modre-Osprian, R.; Welte, S.; Poelzl, G. Evaluation of an Integrated Telemonitoring Surveillance System in Patients with Coronary Heart Disease. Methods Inf Med. 2015;54(5):388-97.

4. Antonicelli RT, P.; Spazzafumo, L.; Gagliardi, C.; Bilo, G.; Valentini, M.; Olivieri, F.; Parati, G. Impact of telemonitoring at home on the management of elderly patients with congestive heart failure. Journal of Telemedicine and Telecare. 2008;14(6):300-5.

5. Boeni FH, K. E.; Arnet, I. Success of a sustained pharmaceutical care service with electronic adherence monitoring in patient with diabetes over 12 months. BMJ Case Reports. 2015;2015.

6. Borah BJQ, Y.; Shah, N. D.; Gleason, P. P. Impact of provider mailings on medication adherence by Medicare Part D members. Healthcare. 2016;4(3):207-16.

7. Brath HM, J.; Kastenbauer, T.; Modre-Osprian, R.; Strohner-Kastenbauer, H.; Schwarz, M.; Kort, W.; Schreier, G. Mobile health (mHealth) based medication adherence measurement - a pilot trial using electronic blisters in diabetes patients. BRITISH JOURNAL OF CLINICAL PHARMACOLOGY. 2013;76:47-55.

8. Chew SL, P. S. M.; Ng, C. J. Usability and Utility of a Mobile App to Improve Medication Adherence Among Ambulatory Care Patients in Malaysia: Qualitative Study. JMIR MHEALTH AND UHEALTH. 2020;8(1).

9. Criner GJC, T.; Hahn, K. A.; Kastango, K.; Eudicone, J.; Gilbert, I. The Impact of Budesonide/Formoterol pMDI Medication Reminders on Adherence in Chronic Obstructive Pulmonary Disease (COPD) Patients: Results of a Randomized, Phase 4, Clinical Study. INTERNATIONAL JOURNAL OF CHRONIC OBSTRUCTIVE PULMONARY DISEASE. 2021;16:563-77.

10. Russell CL, Cetingok M, Hamburger KQ, Owens S, Thompson D, Hathaway D, et al. Medication adherence in older renal transplant recipients. Clin Nurs Res. 2010;19(2):95-112.

11. Desteghe LK, K.; Vijgen, J.; Koopman, P.; Dilling-Boer, D.; Schurmans, J.; Dendale, P.; Heidbuchel, H. The Health Buddies App as a Novel Tool to Improve Adherence and Knowledge in Atrial Fibrillation Patients: A Pilot Study. JMIR MHEALTH AND UHEALTH. 2017;5(7).

12. Desteghe LV, J.; Koopman, P.; Dilling-Boer, D.; Schurmans, J.; Dendale, P.; Heidbuchel, H. Telemonitoring-based feedback improves adherence to non-Vitamin K antagonist oral anticoagulants intake in patients with atrial fibrillation. European Heart Journal. 2018;39(16):1394-403.

13. Dugas MC, K.; Gao, G. G.; Xu, T.; Agarwal, R.; Kruglanski, A. W.; Steinle, N. Individual differences in regulatory mode moderate the effectiveness of a pilot mHealth trial for diabetes management among older veterans. PLOS ONE. 2018;13(3).

14. Elliott RAB, N.; Clifford, S.; Horne, R.; Hartley, E. The cost effectiveness of a telephone-based pharmacy advisory service to improve adherence to newly prescribed medicines. Pharm World Sci. 2008;30(1):17-23.

15. Foreman KFS, K. M.; Le, L. B.; Fisk, E.; Shah, S. M.; Lew, H. C.; Solow, B. K.; Curtis, B. S. Impact of a Text Messaging Pilot Program on Patient Medication Adherence. CLINICAL THERAPEUTICS. 2012;34(5):1084-91.

16. Forestal DAK, T. A.; Peterson, A. M.; Heller, D. A. Initial Medication Adherence in the Elderly Using PACE Claim Reversals: A Pilot Study. JOURNAL OF MANAGED CARE & SPECIALTY PHARMACY. 2016;22(9):1046-50.

17. Goldstein CMG, E. C.; Dolansky, M. A.; Gunstad, J.; Sterns, A.; Redle, J. D.; Josephson, R.; Hughes, J. W. Randomized controlled feasibility trial of two telemedicine medication reminder systems for older adults with heart failure. JOURNAL OF TELEMEDICINE AND TELECARE. 2014;20(6):293-9.

18. Graetz IM, C. N.; Stepanski, E.; Vidal, G. A.; Anderson, J. N.; Schwartzberg, L. S. Use of a web-based app to improve breast cancer symptom management and adherence for aromatase inhibitors: a randomized controlled feasibility trial. Journal of Cancer Survivorship. 2018;12(4):431-40.

19. Guadamuz JSM, C. D.; Choi, S.; Urick, B.; Alexander, G. C.; Qato, D. M. Telepharmacy and medication adherence in urban areas. JOURNAL OF THE AMERICAN PHARMACISTS ASSOCIATION. 2021;61(2):E100-E13.

20. Hale TMJ, K.; Kandola, M. S.; Saldana, F.; Kvedar, J. C. A Remote Medication Monitoring System for Chronic Heart Failure Patients to Reduce Readmissions: A Two-Arm Randomized Pilot Study. JOURNAL OF MEDICAL INTERNET RESEARCH. 2016;18(5).

21. Holender AS, S.; De Simoni, A. Opinions on the use of technology to improve tablet taking in &gt;65-year-old patients on cardiovascular medications. Journal of International Medical Research. 2018;46(7):2754-68.

22. Huang CYN, P. A. A.; Clinciu, D. L.; Hsu, C. K.; Lu, J. C. R.; Yang, H. C.; Wu, C. C.; Tsai, W. C.; Chou, Y. C.; Kuo, T. B. J.; Chang, P. L.; Jian, W. S.; Li, Y. C. J. A personalized medication management platform (PMMP) to improve medication adherence: A randomized control trial. COMPUTER METHODS AND PROGRAMS IN BIOMEDICINE. 2017;140:275-81.

23. Kassavou AAC, C. E.; Chauhan, J.; Brimocombe, J. D.; Bhattacharya, D.; Naughton, F.; Hardeman, W.; Mascolo, C.; Sutton, S. Assessing the acceptability of a text messaging service and smartphone app to support patient adherence to medications prescribed for high blood pressure: A pilot study. Pilot and Feasibility Studies. 2020;6(1).

24. Kim M. Effects of Customized Long-Message Service and Phone-Based Health-Coaching on Elderly People with Hypertension. IRANIAN JOURNAL OF PUBLIC HEALTH. 2019;48(4):655-63.

25. Kobb RH, N.; Lodge, R.; Kline, S. Enhancing elder chronic care through technology and care coordination: report from a pilot. Telemedicine journal and e-health : the official journal of the American Telemedicine Association. 2003;9(2):189-95.

26. Kooy MJvW, B. L. G.; Heerdink, E. R.; de Boer, A.; Bouvy, M. L. Does the use of an electronic reminder device with or without counseling improve adherence to lipid-lowering treatment? The results of a randomized controlled trial. FRONTIERS IN PHARMACOLOGY. 2013;4.

27. Lee J-AE, Lorraine S.; Moore, Alison A.; Juth, Vanessa; Guo, Yuqing; Gago-Masague, Sergio; Lem, Carolyn G.; Nguyen, Michelle; Khatibi, Parmis; Baje, Mark; Amin, Alpesh N. Feasibility Study of a Mobile Health Intervention for Older Adults on Oral Anticoagulation Therapy. Gerontology and Geriatric Medicine. 2016;2.

28. Lien DK, Kiki; Vijgen, Johan; Koopman, Pieter; Dilling-Boer, Dagmara; Schurmans, Joris; Dendale, Paul; Hein, Heidbuchel. The Health Buddies App as a Novel Tool to Improve Adherence and Knowledge in Atrial Fibrillation Patients: A Pilot Study. JMIR mHealth and uHealth. 2017;5(7).

29. Mira JJS, Lorenzo; Nuria, Toro. A Spanish Pillbox App for Elderly Patients Taking Multiple Medications: Randomized Controlled Trial. Journal of Medical Internet Research. 2014;16(4).

30. Mubashir Aslam AA, Ahmad; Chiu, Venus; Kembel, Lorena. Medication adherence support of an in-home electronic medication dispensing system for individuals living with chronic conditions: a pilot randomized controlled trial. BMC Geriatrics. 2021;21:1-16.

31. Ownby RLH, C.; Czaja, S. J. Tailored Information and Automated Reminding to Improve Medication Adherence in Spanish- and English-Speaking Elders Treated for Memory Impairment. CLINICAL GERONTOLOGIST. 2012;35(3):221-38.

32. Panuccio VM, Salvatore; Villa, Antonino; Versace, Maria Carmela; Mercuri, Sergio; Vigni, Maurizio Li; Tripepi, Giovanni; Torino, Claudia. Smit-Ckd: A Mobile App To Improve Adherence To Therapy In Ckd Patients. A Pilot Study. Piscataway: The Institute of Electrical and Electronics Engineers, Inc. (IEEE); 2020. p. 1492-7.

33. Park LG, Howie-Esquivel J, Chung ML, Dracup K. A text messaging intervention to promote medication adherence for patients with coronary heart disease: a randomized controlled trial. Patient Educ Couns. 2014;94(2):261-8.

34. Park LGN, Fion; K. Shim Janet; Elnaggar, Abdelaziz; Villero, Ofelia. Perceptions and experiences of using mobile technology for medication adherence among older adults with coronary heart disease: A qualitative study. Digital Health. 2020;6.

35. Park DY. A Theoretically Informed mHealth Intervention to Improve Medication Adherence by Adults with Chronic Conditions: Technology Acceptance Model-Based Smartphone Medication Reminder App Training Session [Ph.D.]. Ann Arbor: Indiana University - Purdue University Indianapolis; 2019.

36. Park HA, A.; Wang, W.; Roane, T. E. Impact of a telephonic outreach program on medication adherence in Medicare Advantage Prescription Drug (MAPD) plan beneficiaries. Journal of the American Pharmacists Association. 2017;57(1):62-6.e2.

37. Patton DEF, J. J.; Clark, E.; Smith, F.; Cadogan, C. A.; Ryan, C.; Hughes, C. M. A pilot study of the S-MAP (Solutions for Medications Adherence Problems) intervention for older adults prescribed polypharmacy in primary care: Study protocol. Pilot and Feasibility Studies. 2019;5(1).

38. Puig JE, P.; Lluch, T.; Herms, J.; Estany, C.; Bonjoch, A.; Ornelas, A.; Paris, D.; Loste, C.; Sarquella, M.; Clotet, B.; Negredo, E. A Specific Mobile Health Application for Older HIV-Infected Patients: Usability and Patient's Satisfaction. TELEMEDICINE AND E-HEALTH. 2021;27(4):432-40.

39. Qvist IL, J. S.; Søgaard, R.; Lorentzen, V.; Hallas, J.; Frost, L. Randomised trial of telephone counselling to improve participants' adherence to prescribed drugs in a vascular screening trial. Basic and Clinical Pharmacology and Toxicology. 2020;127(6):477-87.

40. Ramachandran BT, C. M.; Wharam, J. F.; Duru, O. K.; Dyer, W. T.; Neugebauer, R. S.; Karter, A. J.; Brown, S. D.; Marshall, C. J.; Wiley, D.; Ross-Degnan, D.; Schmittdiel, J. A. A Randomized Encouragement Trial to Increase Mail Order Pharmacy Use and Medication Adherence in Patients with Diabetes. Journal of General Internal Medicine. 2021;36(1):154-61.

41. Redfern JC, G.; Mulley, J.; Scaria, A.; Neubeck, L.; Hafiz, N.; Pitt, C.; Weir, K.; Forbes, J.; Parker, S.; Bampi, F.; Coenen, A.; Enright, G.; Wong, A. N.; Nguyen, T.; Harris, M.; Zwar, N.; Chow, C. K.; Rodgers, A.; Heeley, E.; Panaretto, K.; Lau, A.; Hayman, N.; Usherwood, T.; Peiris, D. A digital health intervention for cardiovascular disease management in primary care (CONNECT) randomized controlled trial. NPJ DIGITAL MEDICINE. 2020;3(1).

42. Faisal S, Ivo J, McDougall A, Patel T. Stakeholder Feedback of Electronic Medication Adherence Products: Qualitative Analysis. Journal of Medical Internet Research. 2020;22(12):e18074.

43. Schmidt SS, S.; Beil, B.; Patten, M.; Stettin, J. Acceptance of telemonitoring to enhance medication compliance in patients with chronic heart failure. Telemedicine and e-Health. 2008;14(5):426-33.

44. Sheilini MH, H. M.; Prabhu, M. M.; Pai, M. S.; George, A. Impact of multimodal interventions on medication nonadherence among elderly hypertensives: a randomized controlled study. PATIENT PREFERENCE AND ADHERENCE. 2019;13:549-59.

45. Shukla GT, A.; Vishnuprasad, R.; Pradhan, S.; Prakash, M. S. A prospective study to assess the medication adherence pattern among hypertensives and to evaluate the use of cellular phone text messaging as a tool to improve adherence to medications in a tertiary health-care center. INDIAN JOURNAL OF PHARMACOLOGY. 2020;52(4):290-5.

46. Sterns AAS, H. L. Medication Reminding for Older Adults Using Personal Digital Assistants. PROMOTING INDEPENDENCE FOR OLDER PERSONS WITH DISABILITIES. 2006;18:231-4.

47. Toscos TD, M.; Pater, J. A.; Flanagan, M.; Wagner, S.; Coupe, A.; Ahmed, R.; Mirro, M. J. Medication adherence for atrial fibrillation patients: Triangulating measures from a smart pill bottle, e-prescribing software, and patient communication through the electronic health record. JAMIA Open. 2020;3(2):233-42.

48. Varleta PA, M.; Akel, C.; Salinas, C.; Navarrete, C.; Garcia, A.; Echegoyen, C.; Rodriguez, D.; Gramusset, L.; Leon, S.; Cofre, P.; Retamal, R.; Romero, K. Mobile phone text messaging improves antihypertensive drug adherence in the community. JOURNAL OF CLINICAL HYPERTENSION. 2017;19(12):1276-84.

49. Wilkie DJY, Y.; Ezenwa, M. O.; Suarez, M. L.; Dyal, B. W.; Gill, A.; Hipp, T.; Shea, R.; Miller, J.; Frank, K.; Nardi, N.; Murray, M.; Glendenning, J.; Perez, J.; Carrasco, J. D.; Shuey, D.; Angulo, V.; McCurry, T.; Martin, J.; Butler, A.; Wang, Z. J.; Molokie, R. E. A Stepped-Wedge Randomized Controlled Trial: Effects of eHealth Interventions for Pain Control Among Adults With Cancer in Hospice. Journal of Pain and Symptom Management. 2020;59(3):626-36.

50. Wu JYFL, W. Y. S.; Chang, S.; Lee, B.; Zee, B.; Tong, P. C. Y.; Chan, J. C. N. Effectiveness of telephone counselling by a pharmacist in reducing mortality in patients receiving polypharmacy: Randomised controlled trial. British Medical Journal. 2006;333(7567):522-5.

51. Zárate-Bravo EG-V, Juan-Pablo; Torres-Cervantes, Engracia; Ponce, Gisela; Andrade, Ángel G.; Valenzuela-Beltrán, Maribel; Rodríguez, Marcela D. Supporting the Medication Adherence of Older Mexican Adults Through External Cues Provided With Ambient Displays: Feasibility Randomized Controlled Trial. JMIR mHealth and uHealth. 2020;8(3).

52. Zhai PPH, K.; Ji, W. J.; Li, Q.; Shi, L.; Atif, N.; Xu, D.; Li, P. C.; Du, Q. Q.; Fang, Y. Efficacy of Text Messaging and Personal Consultation by Pharmacy Students Among Adults With Hypertension: Randomized Controlled Trial. JOURNAL OF MEDICAL INTERNET RESEARCH. 2020;22(5)
