# Supplementary material for: Factors affecting medication adherence among older adults using tele-pharmacy services: a scoping review
Source: Arch Public Health. 2022 Aug 31;80:199. doi: 10.1186/s13690-022-00960-w (PMC9429665; doi:10.1186/s13690-022-00960-w)
Supplement: Supplementary file 3 — Additional file 3: Table 5. Determinants of elders’ medication adherence in telepharmacy services with references. [file 13690_2022_960_MOESM3_ESM.docx]

**Table 5** Determinants of elders’ medication adherence in telepharmacy services with references

| Main Theme | Sub-Theme | Sub-Theme Categories | Article Counts | Reference |
| --- | --- | --- | --- | --- |
| Application side, back end | | | | |
| Design | Patient -health care professionals Interaction | --- | 25 | (1, 3-5, 12-14, 20, 23, 24, 26, 30-32, 34-37, 39, 42-44, 47, 50, 51) |
|  | Intervention Method | Electronic Medical Device | 13 | (1-3, 5, 7, 9, 10, 29, 30, 33, 43, 44, 47) |
|  |  | Message | 10 | (15, 22-25, 33, 34, 45, 48, 52) |
|  |  | Mobile Application | 16 | (3, 7-9, 13, 23, 27-29, 32, 34, 35, 38, 41, 49, 51) |
|  |  | Postal Mail | 1 | (6) |
|  |  | Web Application | 3 | (18, 32, 37) |
|  |  | Telephone Call | 9 | (12, 14, 25, 31, 36, 39, 40, 44, 50) |
|  |  | e-prescription | 2 | (16, 47) |
|  |  | Remote dispensing by pharmacist | 1 | (19) |
|  | Available user manuals | --- | 4 | (8, 34, 35, 46) |
|  | Usability | --- | 6 | (8, 25, 34, 42, 46, 47) |
|  | Device size | --- | 2 | (42, 47) |
|  | Medicine Storage capacity | --- | 1 | (42) |
| Commercial and Market Aspects | Affordability | --- | 2 | (25, 42) |
|  | Government subsidies | --- | 1 | (42) |
|  | Security and Privacy | --- | 2 | (25, 42) |
| Adherence Measurement | Measurement Method | --- | 2 | (20, 33) |
|  | Measurement Scale | Medication Delay  Non-Adherence level  Non-Compliance level  Day-covered  Forgot  Refill delay  Adherence level  Timing adherence | 1  1  1  4  1  1  N/A  1 | (22)  (14)  (43)  (15, 19, 39, 41)  (22)  (26)  Rest of the Studies  (5) |
| User side, front end | | | | |
| User Health Constraints | Disease | --- | 16 | (1, 6, 7, 9, 15, 18, 19, 27, 28, 32, 38, 40, 45, 48, 49, 52) |
|  | Polypharmacy | --- | 8 | (2, 5-7, 15, 19, 25, 30) |
|  | Age | --- | 3 | (9, 22, 48) |
|  | Disability | --- | 2 | (2, 42) |
|  | Family member/caregiver | --- | 2 | (28, 35) |
|  | Remoteness | --- | 1 | (25) |
| User behavior and perceptions | Baseline Adherence | --- | 3 | (12, 17, 28) |
|  | Patient Behaviour | --- | 3 | (13, 23, 46) |
|  | Patient Preference | --- | 4 | (17, 34, 42, 47) |
|  | Long-term use | --- | 8 | (3, 18, 30, 38-40, 44, 51) |

**References of table 5**

1. Ajit RRF, C. H.; Henson, D. B. Patterns and rate of adherence to glaucoma therapy using an electronic dosing aid. Eye. 2010;24(8):1338-43.

2. Ahmad AC, V.; Arain, M. A. Users' Perceptions of an in-Home Electronic Medication Dispensing System: A Qualitative Study. MEDICAL DEVICES-EVIDENCE AND RESEARCH. 2020;13:31-9.

3. Ammenwerth EW, S.; Baumgartner, C.; Fetz, B.; van der Heidt, A.; Kastner, P.; Modre-Osprian, R.; Welte, S.; Poelzl, G. Evaluation of an Integrated Telemonitoring Surveillance System in Patients with Coronary Heart Disease. Methods Inf Med. 2015;54(5):388-97.

4. Antonicelli RT, P.; Spazzafumo, L.; Gagliardi, C.; Bilo, G.; Valentini, M.; Olivieri, F.; Parati, G. Impact of telemonitoring at home on the management of elderly patients with congestive heart failure. Journal of Telemedicine and Telecare. 2008;14(6):300-5.

5. Boeni FH, K. E.; Arnet, I. Success of a sustained pharmaceutical care service with electronic adherence monitoring in patient with diabetes over 12 months. BMJ Case Reports. 2015;2015.

6. Borah BJQ, Y.; Shah, N. D.; Gleason, P. P. Impact of provider mailings on medication adherence by Medicare Part D members. Healthcare. 2016;4(3):207-16.

7. Brath HM, J.; Kastenbauer, T.; Modre-Osprian, R.; Strohner-Kastenbauer, H.; Schwarz, M.; Kort, W.; Schreier, G. Mobile health (mHealth) based medication adherence measurement - a pilot trial using electronic blisters in diabetes patients. BRITISH JOURNAL OF CLINICAL PHARMACOLOGY. 2013;76:47-55.

8. Chew SL, P. S. M.; Ng, C. J. Usability and Utility of a Mobile App to Improve Medication Adherence Among Ambulatory Care Patients in Malaysia: Qualitative Study. JMIR MHEALTH AND UHEALTH. 2020;8(1).

9. Criner GJC, T.; Hahn, K. A.; Kastango, K.; Eudicone, J.; Gilbert, I. The Impact of Budesonide/Formoterol pMDI Medication Reminders on Adherence in Chronic Obstructive Pulmonary Disease (COPD) Patients: Results of a Randomized, Phase 4, Clinical Study. INTERNATIONAL JOURNAL OF CHRONIC OBSTRUCTIVE PULMONARY DISEASE. 2021;16:563-77.

10. Russell CL, Cetingok M, Hamburger KQ, Owens S, Thompson D, Hathaway D, et al. Medication adherence in older renal transplant recipients. Clin Nurs Res. 2010;19(2):95-112.

11. Desteghe LK, K.; Vijgen, J.; Koopman, P.; Dilling-Boer, D.; Schurmans, J.; Dendale, P.; Heidbuchel, H. The Health Buddies App as a Novel Tool to Improve Adherence and Knowledge in Atrial Fibrillation Patients: A Pilot Study. JMIR MHEALTH AND UHEALTH. 2017;5(7).

12. Desteghe LV, J.; Koopman, P.; Dilling-Boer, D.; Schurmans, J.; Dendale, P.; Heidbuchel, H. Telemonitoring-based feedback improves adherence to non-Vitamin K antagonist oral anticoagulants intake in patients with atrial fibrillation. European Heart Journal. 2018;39(16):1394-403.

13. Dugas MC, K.; Gao, G. G.; Xu, T.; Agarwal, R.; Kruglanski, A. W.; Steinle, N. Individual differences in regulatory mode moderate the effectiveness of a pilot mHealth trial for diabetes management among older veterans. PLOS ONE. 2018;13(3).

14. Elliott RAB, N.; Clifford, S.; Horne, R.; Hartley, E. The cost effectiveness of a telephone-based pharmacy advisory service to improve adherence to newly prescribed medicines. Pharm World Sci. 2008;30(1):17-23.

15. Foreman KFS, K. M.; Le, L. B.; Fisk, E.; Shah, S. M.; Lew, H. C.; Solow, B. K.; Curtis, B. S. Impact of a Text Messaging Pilot Program on Patient Medication Adherence. CLINICAL THERAPEUTICS. 2012;34(5):1084-91.

16. Forestal DAK, T. A.; Peterson, A. M.; Heller, D. A. Initial Medication Adherence in the Elderly Using PACE Claim Reversals: A Pilot Study. JOURNAL OF MANAGED CARE & SPECIALTY PHARMACY. 2016;22(9):1046-50.

17. Goldstein CMG, E. C.; Dolansky, M. A.; Gunstad, J.; Sterns, A.; Redle, J. D.; Josephson, R.; Hughes, J. W. Randomized controlled feasibility trial of two telemedicine medication reminder systems for older adults with heart failure. JOURNAL OF TELEMEDICINE AND TELECARE. 2014;20(6):293-9.

18. Graetz IM, C. N.; Stepanski, E.; Vidal, G. A.; Anderson, J. N.; Schwartzberg, L. S. Use of a web-based app to improve breast cancer symptom management and adherence for aromatase inhibitors: a randomized controlled feasibility trial. Journal of Cancer Survivorship. 2018;12(4):431-40.

19. Guadamuz JSM, C. D.; Choi, S.; Urick, B.; Alexander, G. C.; Qato, D. M. Telepharmacy and medication adherence in urban areas. JOURNAL OF THE AMERICAN PHARMACISTS ASSOCIATION. 2021;61(2):E100-E13.

20. Hale TMJ, K.; Kandola, M. S.; Saldana, F.; Kvedar, J. C. A Remote Medication Monitoring System for Chronic Heart Failure Patients to Reduce Readmissions: A Two-Arm Randomized Pilot Study. JOURNAL OF MEDICAL INTERNET RESEARCH. 2016;18(5).

21. Holender AS, S.; De Simoni, A. Opinions on the use of technology to improve tablet taking in &gt;65-year-old patients on cardiovascular medications. Journal of International Medical Research. 2018;46(7):2754-68.

22. Huang CYN, P. A. A.; Clinciu, D. L.; Hsu, C. K.; Lu, J. C. R.; Yang, H. C.; Wu, C. C.; Tsai, W. C.; Chou, Y. C.; Kuo, T. B. J.; Chang, P. L.; Jian, W. S.; Li, Y. C. J. A personalized medication management platform (PMMP) to improve medication adherence: A randomized control trial. COMPUTER METHODS AND PROGRAMS IN BIOMEDICINE. 2017;140:275-81.

23. Kassavou AAC, C. E.; Chauhan, J.; Brimocombe, J. D.; Bhattacharya, D.; Naughton, F.; Hardeman, W.; Mascolo, C.; Sutton, S. Assessing the acceptability of a text messaging service and smartphone app to support patient adherence to medications prescribed for high blood pressure: A pilot study. Pilot and Feasibility Studies. 2020;6(1).

24. Kim M. Effects of Customized Long-Message Service and Phone-Based Health-Coaching on Elderly People with Hypertension. IRANIAN JOURNAL OF PUBLIC HEALTH. 2019;48(4):655-63.

25. Kobb RH, N.; Lodge, R.; Kline, S. Enhancing elder chronic care through technology and care coordination: report from a pilot. Telemedicine journal and e-health : the official journal of the American Telemedicine Association. 2003;9(2):189-95.

26. Kooy MJvW, B. L. G.; Heerdink, E. R.; de Boer, A.; Bouvy, M. L. Does the use of an electronic reminder device with or without counseling improve adherence to lipid-lowering treatment? The results of a randomized controlled trial. FRONTIERS IN PHARMACOLOGY. 2013;4.

27. Lee J-AE, Lorraine S.; Moore, Alison A.; Juth, Vanessa; Guo, Yuqing; Gago-Masague, Sergio; Lem, Carolyn G.; Nguyen, Michelle; Khatibi, Parmis; Baje, Mark; Amin, Alpesh N. Feasibility Study of a Mobile Health Intervention for Older Adults on Oral Anticoagulation Therapy. Gerontology and Geriatric Medicine. 2016;2.

28. Lien DK, Kiki; Vijgen, Johan; Koopman, Pieter; Dilling-Boer, Dagmara; Schurmans, Joris; Dendale, Paul; Hein, Heidbuchel. The Health Buddies App as a Novel Tool to Improve Adherence and Knowledge in Atrial Fibrillation Patients: A Pilot Study. JMIR mHealth and uHealth. 2017;5(7).

29. Mira JJS, Lorenzo; Nuria, Toro. A Spanish Pillbox App for Elderly Patients Taking Multiple Medications: Randomized Controlled Trial. Journal of Medical Internet Research. 2014;16(4).

30. Mubashir Aslam AA, Ahmad; Chiu, Venus; Kembel, Lorena. Medication adherence support of an in-home electronic medication dispensing system for individuals living with chronic conditions: a pilot randomized controlled trial. BMC Geriatrics. 2021;21:1-16.

31. Ownby RLH, C.; Czaja, S. J. Tailored Information and Automated Reminding to Improve Medication Adherence in Spanish- and English-Speaking Elders Treated for Memory Impairment. CLINICAL GERONTOLOGIST. 2012;35(3):221-38.

32. Panuccio VM, Salvatore; Villa, Antonino; Versace, Maria Carmela; Mercuri, Sergio; Vigni, Maurizio Li; Tripepi, Giovanni; Torino, Claudia. Smit-Ckd: A Mobile App To Improve Adherence To Therapy In Ckd Patients. A Pilot Study. Piscataway: The Institute of Electrical and Electronics Engineers, Inc. (IEEE); 2020. p. 1492-7.

33. Park LG, Howie-Esquivel J, Chung ML, Dracup K. A text messaging intervention to promote medication adherence for patients with coronary heart disease: a randomized controlled trial. Patient Educ Couns. 2014;94(2):261-8.

34. Park LGN, Fion; K. Shim Janet; Elnaggar, Abdelaziz; Villero, Ofelia. Perceptions and experiences of using mobile technology for medication adherence among older adults with coronary heart disease: A qualitative study. Digital Health. 2020;6.

35. Park DY. A Theoretically Informed mHealth Intervention to Improve Medication Adherence by Adults with Chronic Conditions: Technology Acceptance Model-Based Smartphone Medication Reminder App Training Session [Ph.D.]. Ann Arbor: Indiana University - Purdue University Indianapolis; 2019.

36. Park HA, A.; Wang, W.; Roane, T. E. Impact of a telephonic outreach program on medication adherence in Medicare Advantage Prescription Drug (MAPD) plan beneficiaries. Journal of the American Pharmacists Association. 2017;57(1):62-6.e2.

37. Patton DEF, J. J.; Clark, E.; Smith, F.; Cadogan, C. A.; Ryan, C.; Hughes, C. M. A pilot study of the S-MAP (Solutions for Medications Adherence Problems) intervention for older adults prescribed polypharmacy in primary care: Study protocol. Pilot and Feasibility Studies. 2019;5(1).

38. Puig JE, P.; Lluch, T.; Herms, J.; Estany, C.; Bonjoch, A.; Ornelas, A.; Paris, D.; Loste, C.; Sarquella, M.; Clotet, B.; Negredo, E. A Specific Mobile Health Application for Older HIV-Infected Patients: Usability and Patient's Satisfaction. TELEMEDICINE AND E-HEALTH. 2021;27(4):432-40.

39. Qvist IL, J. S.; Søgaard, R.; Lorentzen, V.; Hallas, J.; Frost, L. Randomised trial of telephone counselling to improve participants' adherence to prescribed drugs in a vascular screening trial. Basic and Clinical Pharmacology and Toxicology. 2020;127(6):477-87.

40. Ramachandran BT, C. M.; Wharam, J. F.; Duru, O. K.; Dyer, W. T.; Neugebauer, R. S.; Karter, A. J.; Brown, S. D.; Marshall, C. J.; Wiley, D.; Ross-Degnan, D.; Schmittdiel, J. A. A Randomized Encouragement Trial to Increase Mail Order Pharmacy Use and Medication Adherence in Patients with Diabetes. Journal of General Internal Medicine. 2021;36(1):154-61.

41. Redfern JC, G.; Mulley, J.; Scaria, A.; Neubeck, L.; Hafiz, N.; Pitt, C.; Weir, K.; Forbes, J.; Parker, S.; Bampi, F.; Coenen, A.; Enright, G.; Wong, A. N.; Nguyen, T.; Harris, M.; Zwar, N.; Chow, C. K.; Rodgers, A.; Heeley, E.; Panaretto, K.; Lau, A.; Hayman, N.; Usherwood, T.; Peiris, D. A digital health intervention for cardiovascular disease management in primary care (CONNECT) randomized controlled trial. NPJ DIGITAL MEDICINE. 2020;3(1).

42. Faisal S, Ivo J, McDougall A, Patel T. Stakeholder Feedback of Electronic Medication Adherence Products: Qualitative Analysis. Journal of Medical Internet Research. 2020;22(12):e18074.

43. Schmidt SS, S.; Beil, B.; Patten, M.; Stettin, J. Acceptance of telemonitoring to enhance medication compliance in patients with chronic heart failure. Telemedicine and e-Health. 2008;14(5):426-33.

44. Sheilini MH, H. M.; Prabhu, M. M.; Pai, M. S.; George, A. Impact of multimodal interventions on medication nonadherence among elderly hypertensives: a randomized controlled study. PATIENT PREFERENCE AND ADHERENCE. 2019;13:549-59.

45. Shukla GT, A.; Vishnuprasad, R.; Pradhan, S.; Prakash, M. S. A prospective study to assess the medication adherence pattern among hypertensives and to evaluate the use of cellular phone text messaging as a tool to improve adherence to medications in a tertiary health-care center. INDIAN JOURNAL OF PHARMACOLOGY. 2020;52(4):290-5.

46. Sterns AAS, H. L. Medication Reminding for Older Adults Using Personal Digital Assistants. PROMOTING INDEPENDENCE FOR OLDER PERSONS WITH DISABILITIES. 2006;18:231-4.

47. Toscos TD, M.; Pater, J. A.; Flanagan, M.; Wagner, S.; Coupe, A.; Ahmed, R.; Mirro, M. J. Medication adherence for atrial fibrillation patients: Triangulating measures from a smart pill bottle, e-prescribing software, and patient communication through the electronic health record. JAMIA Open. 2020;3(2):233-42.

48. Varleta PA, M.; Akel, C.; Salinas, C.; Navarrete, C.; Garcia, A.; Echegoyen, C.; Rodriguez, D.; Gramusset, L.; Leon, S.; Cofre, P.; Retamal, R.; Romero, K. Mobile phone text messaging improves antihypertensive drug adherence in the community. JOURNAL OF CLINICAL HYPERTENSION. 2017;19(12):1276-84.

49. Wilkie DJY, Y.; Ezenwa, M. O.; Suarez, M. L.; Dyal, B. W.; Gill, A.; Hipp, T.; Shea, R.; Miller, J.; Frank, K.; Nardi, N.; Murray, M.; Glendenning, J.; Perez, J.; Carrasco, J. D.; Shuey, D.; Angulo, V.; McCurry, T.; Martin, J.; Butler, A.; Wang, Z. J.; Molokie, R. E. A Stepped-Wedge Randomized Controlled Trial: Effects of eHealth Interventions for Pain Control Among Adults With Cancer in Hospice. Journal of Pain and Symptom Management. 2020;59(3):626-36.

50. Wu JYFL, W. Y. S.; Chang, S.; Lee, B.; Zee, B.; Tong, P. C. Y.; Chan, J. C. N. Effectiveness of telephone counselling by a pharmacist in reducing mortality in patients receiving polypharmacy: Randomised controlled trial. British Medical Journal. 2006;333(7567):522-5.

51. Zárate-Bravo EG-V, Juan-Pablo; Torres-Cervantes, Engracia; Ponce, Gisela; Andrade, Ángel G.; Valenzuela-Beltrán, Maribel; Rodríguez, Marcela D. Supporting the Medication Adherence of Older Mexican Adults Through External Cues Provided With Ambient Displays: Feasibility Randomized Controlled Trial. JMIR mHealth and uHealth. 2020;8(3).

52. Zhai PPH, K.; Ji, W. J.; Li, Q.; Shi, L.; Atif, N.; Xu, D.; Li, P. C.; Du, Q. Q.; Fang, Y. Efficacy of Text Messaging and Personal Consultation by Pharmacy Students Among Adults With Hypertension: Randomized Controlled Trial. JOURNAL OF MEDICAL INTERNET RESEARCH. 2020;22(5)
